# On the Effect of Employer Offered Leave of Work on Participation in Continuing Vocational Education and Training – Investigating the Intention-Behavior Relation

**DOI:** 10.3389/fpsyg.2021.807809

**Published:** 2022-01-07

**Authors:** Fabian Rüter

**Affiliations:** German Institute for Adult Education (DIE), Bonn, Germany

**Keywords:** intention-behavior relation, continuing vocational education and training, time, panel analysis, leave of work, hybrid logit models

## Abstract

The availability of time is a deciding factor for participation of adults in continuing vocational education and training (CVET). In view of the importance of time for participation, the present study investigates the impact of employer offered leave of work on employees’ participation behavior in CVET. Leave of work provides a specific timeframe for CVET by enabling the use of working time as learning time. The rationale of the intention-behavior relation as theorized by the theory of planned behavior provides the theoretical framework for the study. The theory allows the integration of individual and contextual factors (e.g., the work environment) in explaining individual behavior and the underpinning decision-making process. The theory conceptualizes time as an element of behavioral control that is required to act on an intention. Behavioral control is theorized to moderate the intention-behavior relation. Two modes of behavioral control are distinguished. We use employer offered leave of work as a proxy for actual behavioral control and the degree of perceived behavioral control regarding the availability of temporal resources to participate in CVET to investigate the theorized moderating role of behavior control on the intention-behavior relation. To test the hypotheses, two waves of panel data from the German National Educational Panel Study (NEPS) are used. Aiming at causal inferences, hybrid logit models are employed. We find that a participation intention is a significant predictor of CVET participation. However, the results provide no evidence regarding the theorized moderating role of actual behavioral control in terms of an employer offered leave of work on the intention-behavior relation. Furthermore, the results provide evidence that the degree of perceived behavioral control regarding the availability of temporal resources to participate in CVET does neither moderate the intention-behavior relation nor is a proxy for actual behavioral control. Finally, we discuss possible future developments of the theory of planned behavior by integrating action-theoretical assumptions from the value-expectancy theory.

## Introduction

Academia, policymakers, and companies have continuously stressed the importance of continuing vocational education and training (CVET) for modern societies (e.g., OECD, 2013; CEDEFOP, 2015; Becker, 2019). CVET is expected to enable economic and social benefits for the society, economy, and individuals, such as safeguarding the labor force, enhancing the economic efficiency and productivity, contributing to the sustainable employability of adults and their social capital as well as opening individual career moves and improving career opportunities (e.g., Barrett and O’Connell, 2001; de Grip and Sauermann, 2013; CEDEFOP, 2015; Ruhose et al., 2020). However, despite significant monetary and non-monetary outcomes, research highlights that opportunities and resources in accessing CVET are unequally distributed depending on characteristics of the individual, the company-side, and the policy context (e.g., Saar and Räis, 2017; Lischewski et al., 2020). In addition, the availability of opportunities does not guarantee participation (Fleuren et al., 2020). Accordingly, Kyndt and Baert (2013) argued that CVET participation cannot be simplified by only fitting supply and demand.

Thus, evidence explaining the (causal) mechanisms that produce disparities as well as success factors for widening access and participation is important (e.g., CEDEFOP, 2014; Siegfried and Berger, 2020; Leyretana and Trinidad, 2021). The most common reasons given by adults for not participating are time-related constraints resulting from work schedules and family responsibilities (e.g., Boeren, 2011; BMBF, 2017). In this regard, participants need to raise temporal resources as an element of indirect costs and monetary recourses as direct costs (Bellmann and Leber, 2019). The perspective on indirect costs builds on the rationale that time is a scarce resource by nature (Klein, 2007). Furthermore, the time required for participation rivals with other time commitments in adults’ private and professional lives (Schmidt-Lauff, 2008; Schmidt-Lauff and Bergamini, 2017). Thereby, opportunity costs for activity participation increase (Klein, 2007), and the availability of temporal resources becomes a deciding factor for participation in educational activities (Rüter et al., 2020). Although academia approaches time-related constraints as the main barrier in accessing educational activities, the corresponding literature is dominated by qualitative studies (e.g., Schmidt-Lauff, 2008; Denninger et al., 2020; Siegfried and Berger, 2020) and multivariate analyses based on cross-sectional survey data (e.g., Boeren, 2011; BMBF, 2017). The respective results provide insights into the subjective perception of time constraints as *situational barriers* (Cross, 1981) and how they may affect the participation decision. However, Rubenson and Desjardins (2009) criticized that these perceptions are subjective thresholds which in turn could be perceived differently. Contrasting the subjective perspective, Boeren (2011) emphasized that time-related constraints due to high working-time can be considered a real and thus objective barrier. However, Rüter et al. (2020) highlighted that causal evidence on the impact of individual time-availabilities on participation in educational activities is scarce and methodologically limited in current research.

An approach to investigate the impact of time on participation is to take actors into account that can affect individual time-availabilities for activity participation. Accordingly, Rüter and Martin (2021) argued that individual time-availabilities that are required to participate in educational activities are not only related to the individual level, but also to societal and organizational levels. A key actor in this consideration is the employer. According to the Adult Education Survey (AES; BMBF, 2019), 54% of 18- to 64-year-old adults in Germany participated in at least one activity of non-formal education in 2018. The largest share constituted work-related training (81%) that took place during paid working time (62%) or paid leave (7%) and was paid by the employer (57%). On the employer-side, a study by BIBB (2020) observed an increasing number of companies that support CVET in Germany (2001: 36%; 2018: 54%). Overall, the employer can provide financial contributions and offer a *leave of work* that provides a specific timeframe for CVET by enabling the use of working time as learning time.

Regarding the significance of time for CVET, the present study investigates how employer offered *leave of work* affects individual participation in CVET. To do so, we build on the theory of planned behavior (TPB, Ajzen, 2012, 2020). We adopt the rationale on how *leave of work* impacts the degree of *actual behavioral control* that an employee has over participating in CVET. This encompasses two aspects: First, we address the questions of how individual time-availabilities affect individual participation behavior and which actors can successfully provide required temporal resources for participation. These two questions are of great importance for academia, policymakers, and practice for providing support for CVET in overcoming barriers and time-related constraints to achieve participation (Boeren, 2011). Second, we aim to put the intention-behavior relation as theorized by the TPB to an empirical test. In this regard, “insufficient time […] may prevent people from acting on their intention” (Ajzen, 2020, p. 2). In a longitudinal design, we draw on data from the German National Educational Panel Study (NEPS; Blossfeld et al., 2011) from two waves. Aiming at causal inferences, we employ hybrid logit models (Allison, 2009; Schunck, 2013; Schunck and Perales, 2017) to estimate the impact of both time-variant and time-invariant variables on CVET participation.

## Patterns of Continuing Vocational Education and Training Participation

Following Lischewski et al. (2020), we can make four overall differentiations in defining CVET. (1) *Purpose*: In comparison to private educational activities, CVET entails any activity with a vocational and work reference. (2) *Segment*: Within CVET, job-related education and employer-sponsored education can be distinguished. (3) *Provision*: Whereas educational activities in the segment of job-related education are initiated by the individual and carried out independently from the workplace, employer-sponsored education is provided by companies and takes (partially) place during paid working time or paid leave. (4) *Degree of Institutionalization*: CVET can be formal, non-formal or informal.

On the national and international comparative level, representative surveys provide insights both into the individual participation and the provision of opportunities and resources by companies. However, such surveys are characterized by a “conceptual diversity of CVET” (Lischewski et al., 2020, p. 3), therewith giving a “scattered impression” (Kyndt and Baert, 2013, p. 307) of CVET participation. However, research has identified patterns of CVET participation (e.g., Grund and Martin, 2012; Becker, 2019; BMBF, 2019; Lischewski et al., 2020). To explain such patterns, scholars build on different theoretical approaches. This includes the theory of human capital (e.g., Becker, 2019), rational choice approaches (e.g., Rüter et al., 2020), expectation-value theory (e.g., Gorges and Kandler, 2012) or the theory of planned behavior (for details: see theoretical framework). In a review of antecedents for formal and informal work-related learning, Kyndt and Baert (2013) identified a total of 117 antecedents of participation covering socio-demographic, personal and job characteristics, characteristics of the learning activity as well as the company and the broader context. This number highlights the multi-layered nature of CVET participation as well as its theoretical and empirical explanation. However, despite the large body of theoretical approaches and empirical findings, one can identify a standard model of determinants in current research: On the individual level, determinants can be broadly classified as socio-demographic, -economic and -cultural factors on one side and psychological factors on the other (Boeren et al., 2010). Key characteristics that contribute to CVET are the individuals’ employment and occupational status, age, gender, level of formal educational attainment, wage, migration background, household characteristics such as marital status, family responsibilities or household income (Lischewski et al., 2020). In addition, scholars investigated a multitude of psychological characteristics including attitude and motivation (e.g., Hurtz and Williams, 2009), intentions (e.g., Kyndt et al., 2011), or personality traits (e.g., Ruhose et al., 2020). On the level of job characteristics and the workplace environment, CVET is associated with factors such as the branch and sector of the company, company size, provided support, opportunities and resources, contract situation, job status and working time (e.g., Grund and Martin, 2012; Kaufmann and Widany, 2013; Becker, 2019). Furthermore, insights from qualitative research in Germany by Denninger et al. (2020) point to positive effects of *leave of work* on individual participation decisions. The review by Kyndt and Baert (2013) revealed that overall, studies focused on socio-demographic variables to explain participation patterns. However, scholars also question the predictive validity of the standard model of social-statistical characteristics. Thus, Walter and Müller (2014) argued that it is not the aforementioned social-statistical characteristics themself that determine the individual decision-making process and predict participation behavior but rather the expected utilities and available resources that are attributed to these characteristics.

## Multi-Layered Backgrounds of Individual Time-Availabilities

The perspective by Walter and Müller (2014) on the significance of resources in explaining participation is particularly evident considering the importance of time as a scarce resource (Klein, 2007) and deciding factor for participation (Rüter et al., 2020). Resulting opportunity costs for activity participation lead to time rivalries “between work, family and recreation on one hand and learning on the other” (Schmidt-Lauff and Bergamini, 2017, p. 157). Schmidt-Lauff (2008) and in later work Schmidt-Lauff and Bergamini (2017) conceptualized individual time-availabilities to result from multi-layered backgrounds. These backgrounds are described by specific time rivalries on the individual level including factors such as age, gender, or employment status (structural time rivalries) as well as different life phases such as career entry, career advancement, starting a family or retirement (biographical time rivalries). In addition, Rüter and Martin (2021) argued that individual time-availabilities are not only related to the individual level, but also to societal and organizational levels. Thus, required temporal resources for participation can arise from parts of the individuals’ time spent at work, for family or recreation or from a combination (Denninger et al., 2020).

## Theoretical Framework

Currently, academia approaches educational activity participation as the result of multi-level interactions between individuals and their decisions to participate, educational providers and the broader societal and policy context (e.g., Boeren et al., 2010). Regarding the individual decision-making process, Baert et al. (2006) theorized several consecutive steps. The process starts from experiencing an (educational) need which in the follow-up should lead to the development of an intention and educational demand. The final step is the participation in an educational activity. In the multi-layered perspective on participation by Boeren et al. (2010), several factors within and between the levels can influence the decision-making process, hindering or facilitating its continuation.

Conceptualizing CVET participation as a process that requires opportunities, and the deliberate investment of resources allows characterizing it as a planned behavior. In a social-psychological perspective, CVET participation can be predicted by a (behavioral) intention (e.g., Maurer et al., 2003; Kyndt and Baert, 2013). To investigate the intention-behavior relation, we build on the theory of planned behavior (TPB; Ajzen, 2012, 2020). Since its formulation, the TPB had a high impact on research in a multitude of behavioral domains (Bosnjak et al., 2020). The TPB is a “*hierarchical (multi-stage) causal model*” (Opp, 2019, p. 68): The model formulates the *behavior* in question at the first stage. The immediate antecedent is the *intention* to perform the behavior at the second stage. Given a sufficient degree of *actual behavior control* (ABC), individuals are expected to act on this intention. Intention itself is a joint function of motivational variables at the third stage: *attitude toward the behavior* (ATT), *subjective norm* attached to the behavior (SN) and *perceived behavioral control* (PBC). ATT, SN and PBC are the respective aggregate of behavioral, normative and control beliefs at the fourth stage. Within the development of the TPB, scholars have applied both modes of *perceived* and *actual behavioral control* differently. PBC was either theorized as a direct determinant of intentions (e.g., Valois et al., 2020), as a moderating variable of the effects of ATT and SN on intention (e.g., La Barbera and Ajzen, 2020, 2021), as a predictor of behavior (e.g., Parkinson et al., 2017) or, as a proxy for ABC (see Fishbein and Ajzen, 2010 for overview). Hence, Bosnjak et al. (2020) as well as La Barbera and Ajzen (2021) recently argued that the theory of planned behavior is still a work in progress and that more research efforts are required to understand human behavior and its determinants including the concepts of actual and perceived behavioral control.

### The Intention-Behavior Relation

The TPB approaches intentions as the best single predictor of behavior (Fishbein and Ajzen, 2010). Evidence from meta-analytical reviews in a multitude of behavioral domains revealed that this assumption holds true (e.g., Armitage and Conner, 2001; Sheeran, 2002; McDermott et al., 2015; Nguyen et al., 2019). In the field of adult learning and education, scholars found a positive relation between intention and participation (e.g., Maurer et al., 2003; Kyndt et al., 2011). However, in a meta-analytical approach, Sheeran (2002) concluded that overall, intentions are realized only half of the time. This discrepancy raises questions of contexts and conditions that underlie a successful implementation of an intention. According to the TPB, intentions predict behavior to the extent that an individual is capable of performing the behavior, thus has sufficient *actual behavioral control* (ABC). Therewith, the TPB considers that a lack of required skills, information, resources, opportunities, and abilities as well as barriers or constraints potentially prevent individuals from acting on their intentions (Ajzen, 2020). Whereas PBC refers to the individual’s perception of the degree of behavioral control (Fishbein and Ajzen, 2010), ABC is a “link to reality” (Opp, 2019, p. 92). In this regard, results of the AES in Germany (BMBF, 2017) highlighted that respondents with unfulfilled intentions stated time-related constraints resulting from work schedules and family responsibilities as the main reasons for non-participation. Thus, the availability of temporal resources becomes a deciding factor especially for those who intend to participate. In a study based on Flemish AES data, Boeren (2011) conceptualized the gap between an intention to participate in formal or non-formal education and participation by means of barriers. The results revealed that up to 30% of the surveyed *Inclined Abstainer*s with unfulfilled intentions to participate stated conflicts between the time required to participate and commitments in their private and professional lives.

Given the importance of ABC as a condition of behavior, Sheeran and Webb (2016) criticized, that much of the studies testing the basic assumptions of TPB do not test the intention-behavior relation. In fact, only few studies have explicitly investigated the theoretical mechanisms underpinning the intention-behavior relation. Three reasons are considered in current literature: First, at “its core, the TPB is concerned with the prediction of intentions” (Ajzen, 2011, p. 1115). However, the TPB is a heuristic theory including assumptions on behavior and its predictive antecedents. Second, because knowledge of ABC is not available for most behaviors due to measurement issues, Fishbein and Ajzen (2010) highlighted that researcher often use PBC as a proxy. Respectively, studies found only weak or no significant interaction effects (Yang-Wallentin et al., 2004). Third, La Barbera and Ajzen (2020) argued that such findings result from methodological difficulties or insufficient variance in the predicator and moderator variables.

The continuing development of the TPB challenges scholars to continuously develop new research strategies and methodological approaches to submit the theoretical assumptions to empirical tests (Bosnjak et al., 2020). This includes testing the intention-behavior relation (Sheeran and Webb, 2016) and generating evidence on how policymakers, educational institutions or the employer can support adults in overcoming barriers and time-related constraints to achieve participation (Boeren, 2011). Furthermore, testing the basic assumptions of the TPB includes assessing the assumptions of causal influence among the different stages of the theory (Sussman and Gifford, 2019). In this regard, Ajzen (2012) pointed out, that most empirical evidence on the intention-behavior relation is of correlational nature.

## The Present Research

Although current academia argues for the significance of individual time-availabilities for participation in educational activities (e.g., Schmidt-Lauff and Bergamini, 2017), empirical studies aiming at causal inferences on such effects are still rare (Rüter et al., 2020). This insight follows an earlier call for “further study of the relationship between the motivation to participate in CVET, the ways in which this is translated into real participation, and the impact of time as a resource in this process, both independently and in relation with other resources” (Sellin and Elson-Rogers, 2003, p. 29). Hence, we contribute to current research literature on the impact of time as a recourse on educational activity participation and its underpinning decision-making process by investigating the effect of employer offered leave of work on CVET participation. The TPB (Ajzen, 2012, 2020) theorizes the intention to participate to be the immediate antecedent of CVET participation. In current literature, there is ample evidence that this assumption holds true (see Kyndt and Baert, 2013 for review). Thus, our first hypothesis considers the assumption that CVET participation is an intentional behavior:

(H1) The intention to participate in CVET predicts actual CVET participation.

Further hypotheses focus on the TPB’s concept of behavioral control. The revised model by Ajzen (2012, 2020) distinguishes between a *perceived* (PBC) and an *actual* (ABC) mode of behavioral control. Associated with the two modes are assumptions about causal influences on different stages of the theoretical model. Whereas PBC is theorized to influence the formulation of an intention, ABC is theorized to moderate the intention-behavior relation (Ajzen, 2020). This means, that the effect of intention on behavior varies according to contextual conditions, which the TPB conceptualizes as ABC. With our study, we focus on the availability of temporal recourses in terms of employer offered *leave of work*. Based on the TPB, we adopt the rationale on how a *leave of work* impacts the ABC that an employee has over participating in CVET. Therefore, employees who were offered a *leave of work* have a higher ABC. Thus, we hypothesize a positive effect of *leave of work* on the intention-behavior relation and subsequently on CVET participation. Based on the assumption that the “greater the actor’s control over the behavior, the more likely it is that the intention will be carried out” (Ajzen, 2020, p. 2), we derive our second hypothesis as follows:

(H2) Employer offered *leave of work* increases the likelihood that employees who intend to participate in CVET actually participate in CVET.

Conversely, a vast majority of research on the impact of time on participation approaches the subjective understanding and interpretation of time-related constraints and time-availabilities as the deciding factor (e.g., Schmidt-Lauff, 2008; Denninger et al., 2020). Accordingly, current literature emphasizes that the intention-behavior relation should rather be moderated by PBC than ABC. This assumption also relates to prior models of the TPB and the use of PBC as a proxy for ABC (Fishbein and Ajzen, 2010). In terms of the TPB and the mode of perceived behavioral control, availability or lack of time is regarded as a control factor that can facilitate or impede the performance of a behavior (Ajzen, 2020). In line with the current literature’s perspective on the deciding effect of the subjective perception of time-related constraints and time-availabilities on participation, our third hypothesis is:

(H3) The degree of *perceived behavioral control* regarding the availability of temporal resources to participate in CVET moderates the effect of an intention to participate in CVET on actual CVET participation.

Furthermore, the theoretical framework of the TPB allows us to investigate whether there is a difference between the two modes of *perceived* and *actual* behavioral control in moderating the effect of intention on CVET participation. Based on the revised model, PBC has no independent moderating effect on the intention-behavior relation. Therefore, the degree of *perceived behavioral control* regarding the availability of temporal resources to participate can only have a moderating effect on the intention-behavior relation for employees who were offered a *leave of work* in terms of ABC. Based on the assumption that PBC has no independent effect of the intention-behavior relation and can only be used as a proxy for ABC, our fourth hypothesis is:

(H4) The degree of *perceived behavioral control* regarding the availability of temporal resources to participate in CVET only moderates the effect of an intention to participate in CVET on actual CVET participation for employees who were offered a *leave of work* to participate in CVET.

## Materials and Methods

### Data Description

To test the hypotheses, we use panel data from the German National Educational Panel Study (NEPS; Blossfeld et al., 2011). The NEPS is an annual multi-cohort panel study in Germany, in which since 2007 approximately 60,000 individuals in eleven waves are questioned about their educational behavior as well as their socioeconomic and -demographic background. We use data from the starting cohort 6 (Adults). The NEPS data are particularly suited for our study for two reasons: First, the NEPS covers all variables required to test our hypotheses regarding the intention-behavior relation derived from the TPB. The intention item was measures in waves 4 (2011/2012) and 8 (2015/2016). Therefore, we use two waves of panel data from the NEPS. Second, the NEPS data include detailed information on educational and employment trajectories as well as participation in educational activities. In the following, we describe the process of creating our analytical samples as well as the measures and all variables used in the models.

### Respondents

In our analytical sample, we only include respondents who were interviewed in both waves (4 and 8), who were employed in both waves and who gave consistent information on whether they were offered a leave of work to participate in CVET by their employer. Given these restrictions, the analytical sample was reduced to *n* = 1,894 respondents and *N* = 3,788 observations. The analytical sample consists of *n* = 1,175 respondents that were offered a leave of work by their employer and *n* = 719 respondents that were not offered a leave of work.

### Measures

Based on our hypotheses derived from the theory of planned behavior, there are four main variables relevant for our study. The *intention* to participate in CVET (predictor variable of behavior); *participation* in CVET (outcome variable); employer offered leave of work as a proxy for *actual behavioral control* (ABC) and the degree of *perceived behavioral control* (PBC) regarding the availability of temporal recourses to participate in CVET (moderating variables).

#### Intention

The theory of planned behavior theorizes the intention as the immediate antecedent and predictor of a behavior. The *intention* to participate in CVET is measured with the item “Do you intend to take this type of course or training program in the next 12 months?”. The item is measured in waves 4 and 8 as a binary variable (1 “Yes”; 2 “No”). This allows us to estimate both intra-individual and inter-individual effects of intention on CVET participation.

#### Participation in Continuing Vocational Education and Training

To investigate whether an intention to participate in CVET was implemented, it is required to operationalize the individual participation behavior in CVET. In the NEPS, the respondents’ *participation* in educational activities is measured in each wave by asking if a respondent has attended any courses since the last interview. For reported courses, different information are surveyed. Participation in CVET is measured in every wave for educational activities (courses) that were reported by a respondent with the item “Did you attend this course primarily for professional reasons or rather out of personal interest?”. The item is measured as a categorial variable (1 “For professional reasons”; 2 “For personal reasons”; 3 “Both professional and personal reasons”). We define all attended courses as participation in CVET that were reportedly attended for professional or for both professional and personal reasons. However, for each respondent, the NEPS only measures this information for two courses which are randomly selected from the total number of courses reported. Because CVET participation is the outcome variable in our study, we need the information about the reason of attending a course for every reported course. Therefore, we build on an approach proposed by Ebner and Ehlert (2018) and impute the missing information. For this, we use the logistic regression imputation method for binary variables by Rubin (1987). We use both course-level information (e.g., duration, content) as well as control variables of our empirical models as imputation variables (10 imputations). The intention item measured in waves 4 and 8 defines a timeframe of 12 months after the interview date in which participation in CVET is defined as intended. Therefore, we measure CVET participation in the timeframe of up to 12 months after the respondents were interviewed. We aggregate the information regarding the reasons for attending all reported courses in the 12-month period after the interview date into a new variable that indicates whether at least one of the reported courses per respondent and wave was attended for professional or for both professional and personal reasons. This allows us to identify whether an intention to participate in CVET was implemented into actual CVET participation or not.

#### Leave of Work as a Proxy for Actual Behavioral Control

The first variable that is assumed by the theory of planned behavior to moderate the effect of intention on behavior is actual behavioral control (ABC). In our study, we use employer offered leave of work as a proxy for ABC. In the NEPS, employer offered *leave of work* is measured for employment episodes of a respondent with the item “Has your current employer offered you a leave of work to attend training programs and courses?” or “Did your former employer offer you a leave of work to attend training programs and courses?”. The item is measured as a dichotomous variable (1 “Yes”; 2 “No”). Since the *leave of work* item is not consistently measured for every employment episode that a respondent has reported, we are not able to reliably identify intra-individual changes regarding employer offered *leave of work* between wave 4 and 8. Hence, we use *leave of work* as a time-invariant variable to observe inter-individual differences. Respondents who reported changes regarding employer offered *leave of work* between waves 4 and 8 are excluded from our analytical sample. In case of secondary employment, i.e., when several employment episodes take place at the same time in waves 4 and 8, it is sufficient if *leave of work* is offered by the employer in one of the employment episodes.

#### Degree of Perceived Behavioral Control

The second variable that is assumed by the theory of planned behavior to moderate the effect of intention on behavior is the degree of perceived behavioral control (PBC). In the NEPS, the degree of perceived behavioral control (PBC) regarding the availability of temporal resources to participate in CVET is measured with the item “Attending would take too much time.” The item is measured on a 5-point Likert scale ranging from 1 (“I completely agree”) to 5 (“I don’t agree at all”) in waves 4 and 8. This allows us to observe both intra-individual and inter-individual changes.

#### Demographics and Further Variables

In the NEPS, different variables on the individual-level and the reported employment episodes are measured. Different time-invariant variables (e.g., gender) were collected in the respondent’s first interview. Different time-variant variables (e.g., household net income) are collected in each wave. The NEPS data include detailed information for employment episodes that a respondent has reported (e.g., branch, company size) and regarding the support provided for CVET participation (e.g., financial support). Like the *leave of work* item, we are not able to reliably identify intra-individual changes regarding the support for participation in CVET for every reported employment episode. Hence, we use such items as time-invariant variables to observe inter-individual differences. Respondents who reported changes regarding these variables between waves 4 and 8 are excluded from our analytical sample. In case of secondary employment in wave 4 or wave 8, it is sufficient if support for CVET participation is provided by the employer in one of the employment episodes.

### Estimation Strategy

Our hypotheses target potential effects of employer offered *leave of work* on CVET participation and theoretical assumptions formulated by the TPB regarding the intention-behavior relation. Given that our hypotheses refer to both between-individual and within-individual effects on CVET participation, we employ hybrid logit models (Allison, 2009; Schunck, 2013; Schunck and Perales, 2017). Generally speaking, hybrid models combine random-effects models with fixed-effects models and thus allow estimating and comparing effects on the within-person level and on the between-person level in one single model (Allison, 2009). Applied to our research questions, hybrid logit models allow estimating the impact of both time-variant variables (e.g., intention) and time-invariant variables (e.g., *leave of work*) on a dichotomous dependent variable (participation in CVET) while including different interaction terms (i.e., intention and leave of work) and controlling for further variables (i.e., age, gender, educational background, net household income). This is accomplished in a first step by decomposing each independent time-varying variable into a between-individual component (individual-specific mean) and a within-individual component (deviation from the individual-specific mean). In a second step, we estimate a random-effects model that includes both between-individual and within-individual components (Allison, 2009). Regarding Hypothesis 1, we specify the hybrid logit model as follows:


(1) $$\begin{array}{c} logit\left(y_{it}\right)=\beta_{0}+\sum_{a=1}^{a=39}\beta_{Wa}\left(x_{ait}-{\bar{x}}_{ai}\right)+\sum_{a=1}^{a=39}\beta_{Ba}{\bar{x}}_{ai}+\\ \;\sum_{a=1}^{a=10}\gamma_{a}c_{ai}+u_{i}+\varepsilon_{it} \end{array}$$

Here, *y*_*it*_ denotes the dependent variable describing whether respondent *i* participated in at least one activity of CVET at time *t*. Then, β_0_ is the constant. *x*_1_ to *x*_39_ are the values of independent time-varying variables in the model. This includes the intention to participate in CVET (*x*_1_), respondents age (*x*_2_), net household income (*x*_3_ − *x*_9_), whether there are children in the household (*x*_10_), marital status (*x*_11_ − *x*_13_), how well informed the respondent is about CVET programs and courses (*x*_14_), occupational area (*x*_15_ − *x*_22_) and professional status (*x*_23_ − *x*_24_) for the employment episode with the highest working hours (in case of secondary employment), total working time per week (*x*_25_), company size for the employment episode with the highest working hours (in case of secondary employment) (*x*_26_ − *x*_35_), whether the respondent is working in a knowledge-intensive economic sector (*x*_36_), secondary employment (*x*_37_), the degree of perceived behavioral control regarding the availability of monetary resources to participate in CVET(*x*_38_), and the degree of perceived behavioral control regarding the availability of temporal resources to participate in CVET (*x*_39_). We include both the person-specific mean (${\bar{x}}_{ai}$) and the deviation from the person-specific mean ($x_{ait}-{\bar{x}}_{ai}$) among the model covariates. Then, β_*B*_ give the between-person effects and β_*W*_ give the within-person effects (Schunck and Perales, 2017). *c*_1_ to *c*_10_ are the values of the independent time-constant variables in the model. This includes the respondents’ gender (*c*_1_), migration background (*c*_2_), years of education (*c*_3_), whether the respondent changed the company between wave 4 and wave 8 (*c*_4_), whether there is a company agreement about further education (*c*_5_), whether a company finances or provides classes or training courses (*c*_6_), whether an employer offered financial support (*c*_7_), whether a company has an education planning on a regular basis for employees (*c*_8_), whether there is a staff member, unit, or department responsible for training or continuing education in a company (*c*_9_), and whether an employer offered a *leave of work* to attend training programs and courses (*c*_10_). *u_i_* is the level-two error and random intercept. ε_*it*_ is the level-one error. Hence, there is a different ε_*it*_ for each individual respondent *i* at each point in time *t*, but *u_i_* only varies across individuals and not over time (Allison, 2009).

Regarding Hypothesis 2, we include an interaction term of intention (*x*_1_) and *leave of work* (*c*_10_) into the model. To do so, we first generate the interaction variable *x*_1*it*_*c*_10*i*_. Following the rationale of the hybrid model approach, we then enter both the mean $\bar{x_{1i}c_{10i}}$ and deviation score $x_{1it}c_{10i}-\bar{x_{1i}c_{10i}}$ of the interaction variable into the model. We specify the hybrid logit model as follows:


(2) $$\begin{array}{c} logit\left(y_{it}\right)=\beta_{0}+\sum_{a=1}^{a=39}\beta_{Wa}\left(x_{ait}-{\bar{x}}_{ai}\right)+\\ \;\beta_{W}\left(x_{1it}c_{10i}-\bar{x_{1i}c_{10i}}\right)+\sum_{a=1}^{a=39}\beta_{Ba}{\bar{x}}_{ai}+\\ \;\beta_{B}\bar{x_{1i}c_{10i}}+\sum_{a=1}^{a=10}\gamma_{a}c_{ai}+u_{i}+\varepsilon_{it} \end{array}$$

Regarding Hypothesis 3, we include an interaction term of intention (*x*_1_) and the degree of perceived behavioral control regarding the availability of temporal resources to participate in CVET (*x*_39_) into the model. Again, we first generate the interaction variable *x*_1*it*_*x*_39*it*_ and then enter both the mean $\bar{x_{1i}x_{39i}}$ and deviation score $x_{1it}x_{39it}-\bar{x_{1i}x_{39i}}$ of the interaction variable into the model. We specify the hybrid logit model as follows:


(3) $$\begin{array}{c} logit\left(y_{it}\right)=\beta_{0}+\sum_{a=1}^{a=39}\beta_{Wa}\left(x_{ait}-{\bar{x}}_{ai}\right)+\\ \;\beta_{W}\left(x_{1it}x_{39it}-\bar{x_{1i}x_{39i}}\right)+\sum_{a=1}^{a=39}\beta_{Ba}{\bar{x}}_{ait}+\\ \;\beta_{B}\bar{x_{1i}x_{39i}}+\sum_{a=1}^{a=10}\gamma_{a}c_{ai}+u_{i}+\varepsilon_{it} \end{array}$$

Regarding Hypothesis 4, we fit the hybrid logit model as before in equation 3 but separately estimate the model for those individuals who were offered a *leave a work* and for those who were not offered a *leave of work*.


(4) $$\begin{array}{c} logit\left(y_{it}\right)=\beta_{0}+\sum_{a=1}^{a=39}\beta_{Wa}\left(x_{ait}-{\bar{x}}_{ai}\right)+\\ \;\beta_{W}\left(x_{1it}x_{39it}-\bar{x_{1i}x_{39i}}\right)+\sum_{a=1}^{a=39}\beta_{Ba}{\bar{x}}_{ait}+\\ \;\beta_{B}\bar{x_{1i}x_{39i}}+\sum_{a=1}^{a=9}\gamma_{a}c_{ai}+u_{i}+\varepsilon_{it},D=\left\{0,1\right\} \end{array}$$

Here, we exclude the *leave of work* item *c*_10_. *D* = {0, 1} is the indicator of whether an individual was offered a *leave of work* or not.

The study was preregistered on OSF^1^. All analyses were performed with Stata (version 15.1). The corresponding do-file can be found on OSF^2^.

## Results

### Descriptive Statistics

The descriptive statistics of the analytical sample and subsamples are displayed in Table 1. We also estimated the variance inflation factor (VIF) for each independent variable used in the models as a diagnostic for multicollinearity. The results reveal that multicollinearity is not a problem. Variables with high a VIF (VIF > 2.50) are dummy variables representing a categorical variable. The results can be found on OSF (see text footnote 2).

**TABLE 1:** Descriptive statistics of the analytical samples.

	Total analytical sample *N* = 3,788 *n* = 1,894			Subsample Hypothesis 4 (No Leave of work) *N* = 1,438 *n* = 719			Subsample Hypothesis 4 (Leave of work) *N* = 2,350 *n* = 1,175		
			
	*M*	SD	mix./max.	*M*	SD	mix./max.	*M*	SD	mix./max.
Age (in years)	49.31	8.08	25.08/70.33	49.43	8.18	26.08/69.17	49.23	8.01	25.08/70.33
Years of education	14.34	2.22	9/18	13.76	2.19	9/18	14.7	2.16	9/18
Total working time per week in hours	38.19	12.08	0/90	36.37	12.93	0/90	39.31	11.39	0/90
Information – courses and training	3.2	1.03	1/5	2.76	1.02	1/5	3.47	0.94	1/5
Time costs of courses and training (PBC)	3.27	1.24	1/5	3.08	1.27	1/5	3.39	1.22	1/5
Direct costs of courses and training (PBC)	3.78	1.19	1/5	3.55	1.24	1/5	3.92	1.13	1/5

	**% overall**	**% between**	**% within**	**% overall**	**% between**	**% within**	**% overall**	**% between**	**% within**

**Participation in CVET^a^**									
No^b^	60.4	74.53	79.96	75.06	86.16	85.98	51	67.39	75.23
Yes	39.6	55.35	73.01	24.94	37.99	68.22	49	66	74.7
**Intention**									
No	58.1	74.39	78.11	76.08	88.6	85.87	47.11	65.7	71.7
Yes	41.9	58.18	72.01	23.92	36.44	65.65	52.89	71.49	73.99
**Gender**									
(1) Male	51.32	51.32	100	49.93	49.93	100	52.17	52.17	100
(2) Female	48.68	48.68	100	50.07	50.07	100	47.83	47.83	100
**Net household income**									
(1)≤999 €	0.69	1.21	30.52	1.32	2.36	55.88	0.3	0.51	58.33
(2) 1,000 – 1,499 €	3.06	4.86	63.04	4.17	6.68	62.5	2.38	3.74	63.64
(3) 1,500 – 1,999 €	5.83	9.24	63.14	7.23	11.54	62.65	4.98	7.83	63.59
(4) 2,000 – 2,499 €	9.32	15.73	59.23	12.87	21.7	59.29	7.15	12.09	59.15
(5) 2,500 – 2,999 €	11.51	19.27	59.73	13.7	22.95	59.7	10.17	17.02	59.75
(6) 3,000 – 3,999 €	26.14	39.49	66.18	27.96	41.86	66.78	25.02	38.04	65.77
(7) 4,000 – 4,999 €	20.86	33.32	62.6	18.01	29.49	61.08	22.6	35.66	63.37
(8) ≥5,000 €	22.6	30.52	74.05	14.74	21.14	69.74	27.4	36.26	75.59
**Marital status**									
(1) Married/in registered partnership	75.48	78.41	96.26	74.27	77.33	96.04	76.21	79.06	96.39
(2) Divorced	8.21	9.82	83.6	8.83	10.99	80.38	7.83	9.11	85.98
(3) Widowed	2.09	2.32	89.77	2.92	3.06	95.45	1.57	1.87	84.09
(4) Single	14.23	15.42	92.29	13.98	14.88	93.93	14.38	15.74	91.35
**Children**									
(1) No children	44.77	51.8	86.44	46.31	52.29	88.56	43.83	51.49	85.12
(2) Having children	55.23	62.25	88.72	53.69	59.67	89.98	56.17	63.83	88
**Migration background**									
(1) Natives	84.85	84.85	100	81.78	81.78	100	86.72	86.72	100
(2) Immigrants	15.15	15.15	100	18.22	18.22	100	13.28	13.28	100
**Occupational areas (1-digit KldB 2010)**									
(1) Occupations in agriculture, forestry, farming, and gardening	0.87	0.9	97.06	1.18	1.25	94.44	0.68	0.68	100
(2) Occupations in production of raw materials and goods, and manufacturing	18.45	18.8	98.17	24.48	25.03	97.78	14.77	14.98	98.58
(3) Occupations in construction, architecture, surveying and technical building services	4.36	4.44	98.21	5.42	5.56	97.5	3.7	3.74	98.86
(4) Occupations in natural sciences, geography and informatics	6.05	6.23	97.03	3.76	3.89	96.43	7.45	7.66	97.22
(5) Occupations in traffic, logistics, safety, and security	8.42	8.55	98.46	12.93	13.21	97.89	5.66	5.7	99.25
(6) Occupations in commercial services, trading, sales, the hotel business and tourism	8.92	9.35	95.48	12.52	13.07	95.74	6.72	7.06	95.18
(7) Occupations in business organization, accounting, law and administration	25.5	25.98	98.17	19.82	20.31	97.6	28.98	29.45	98.41
(8) Occupations in health care, the social sector, teaching And education	24.87	25.34	98.13	16.76	16.97	98.77	29.83	30.47	97.91
(9) Occupations in philology, literature, humanities, social sciences, economics, media, art, culture, and design	2.56	2.69	95.1	3.13	3.2	97.83	2.21	2.38	92.86
**Professional status**									
(1) Worker	16.79	17.05	98.45	29.76	30.32	98.17	8.85	8.94	99.05
(2) Employee, also employee in public service	73.47	73.76	99.61	65.23	65.79	99.15	78.51	78.64	99.84
(3) Civil servant, also judge, excluding soldiers	9.74	9.77	99.73	5.01	5.01	100	12.64	12.68	99.66
**Knowledge-intensive economic sector**									
No	62.38	63.25	98.62	67.52	68.43	98.68	59.23	60.09	98.58
Yes	37.62	38.49	97.74	32.48	33.38	97.29	40.77	41.62	97.96
**Company size (Number of Employees)**									
(1) <5	4.41	4.96	88.83	7.51	8.48	88.52	2.51	2.81	89.39
(2) 5 – 9	8.21	8.92	92.01	9.46	10.71	88.31	7.45	7.83	95.11
(3) 10 – 19	9.98	10.67	93.56	10.78	11.96	90.12	9.49	9.87	96.12
(4) 20 – 49	16.05	16.79	95.6	18.85	19.75	95.42	14.34	14.98	95.74
(5) 50 – 99	11.22	11.93	94.03	12.24	13.07	93.62	10.6	11.23	94.32
(6) 100 – 199	11.56	12.14	95.22	11.82	12.38	95.51	11.4	12	95.04
(7) 200 – 249	3.99	4.12	96.79	3.89	3.89	100	4.04	4.26	95
(8) 250 – 499	9.64	10.03	96.05	9.67	10.01	96.53	9.62	10.04	95.76
(9) 500 – 999	8	8.55	93.52	5.49	5.7	96.34	9.53	10.3	92.56
(10) 1,000 – 1,999	5.39	5.81	92.73	3.76	4.17	90	6.38	6.81	93.75
(11) ≥2,000	11.56	11.99	96.48	6.54	6.82	95.92	14.64	15.15	96.63
**Secondary employment**									
No	89.47	92.93	96.28	90.13	93.32	96.57	89.06	92.68	96.1
Yes	10.53	13.99	75.28	9.87	13.07	75.53	10.94	14.55	75.15
**Change of company**									
No	93.82	93.82	100	92.91	92.91	100	94.38	94.38	100
Yes	6.18	6.18	100	7.09	7.09	100	5.62	5.62	100
**Offered leave of work**									
No	37.96	37.96	100	100	100	100	−	−	−
Yes	62.04	62.04	100	−	−	−	100	100	100
**Company agreement**									
No	43.72	43.72	100	64.95	64.95	100	30.72	30.72	100
Yes	56.28	56.28	100	35.05	35.05	100	69.28	69.28	100
**Financing**									
No	18.53	18.53	100	43.12	43.12	100	3.49	3.49	100
Yes	81.47	81.47	100	56.88	56.88	100	96.51	96.51	100
**Offered financial support**									
No	39.12	39.12	100	78.86	78.86	100	14.81	14.81	100
Yes	60.88	60.88	100	21.14	21.14	100	85.19	85.19	100
**Planning**									
No	38.07	38.07	100	62.31	62.31	100	23.23	23.23	100
Yes	61.93	61.93	100	37.69	37.69	100	76.77	76.77	100
**Responsible person**									
No	35.16	35.16	100	56.61	56.61	100	22.04	22.04	100
Yes	64.84	64.84	100	43.39	43.39	100	77.96	77.96	100

*M, Mean. SD, standard deviation.*

*^a^Reflects the percentage of respondents who reported at least one course in CVET (dummy variable). Here, we report the descriptive statistics for the original sample, excluding the imputed values for the CVET variable.*

*^b^Indicates whether either no course was attended or whether the course was attended for private reasons.*

### Hybrid Models

To test our hypotheses, we estimated five different hybrid logit models for CVET participation. In the following, we present both within-individual and between-individual effects for each model. We report all estimated coefficients transformed to odds ratios by exponentiating the regression coefficients. Standard errors and confidence intervals are transformed accordingly. Applying a random-effects logistic regression model “fits *subject-specific* or *conditional* probabilities for the individual” (Rabe-Hesketh and Skrondal, 2012, p. 529) respondents given the subject-specific random intercept and covariates in the model. Accordingly, obtained odds ratios from these models are interpreted as subject-specific odds ratios. Thus, we interpret the exponentiated regression coefficients as conditional odds.

Regarding Hypothesis 1, we tested whether CVET participation is an intentional behavior. The results displayed in Table 2 reveal both significant within and between effects of an intention on CVET participation. Within-individual, the odds ratio for CVET participation is 1.713 (*p* < 0.001). This means that the conditional odds of CVET participation for an individual that formulates an intention are 1.713 times those of an individual that did not formulate an intention to participate. Between-individual, the conditional odds of CVET participation for those who intended to participate is four times (OR = 4.066, *p* < 0.001) that of those who did not intended to participate.

**TABLE 2:** Results (Hypothesis 1).

	*N* = 3,788 *n* = 1,894			
	Between-individual		Within-individual	
	OR	CI	OR	CI
Intention	4.066*** (0.579)	[3.08, 5.37]	1.713*** (0.239)	[1.30, 2.25]
Age	0.998 (0.007)	[0.98, 1.01]	0.886*** (0.021)	[0.85, 0.93]
**Net household income(ref. 3,000 – 3,999 €)**				
(1) ≤999 €	0.376 (0.343)	[0.06, 2.25]	1.324 (1.305)	[0.19, 9.15]
(2) 1,000 – 1,499 €	1.249 (0.463)	[0.60, 2.58]	1.076 (0.571)	[0.38, 3.04]
(3) 1,500 – 1,999 €	1.291 (0.356)	[0.75, 2.22]	1.125 (0.436)	[0.53, 2.41]
(4) 2,000 – 2,499 €	1.331 (0.314)	[0.84, 2.11]	1.187 (0.328)	[0.69, 2.04]
(5) 2,500 – 2,999 €	0.710 (0.159)	[0.46, 1.10]	0.978 (0.244)	[0.60, 1.60]
(7) 4,000 – 4,999 €	1.076 (0.191)	[0.76, 1.52]	0.696 (0.139)	[0.47, 1.03]
(8) ≥5,000 €	0.828 (0.139)	[0.60, 1.15]	0.624 (0.157)	[0.38, 1.02]
Children	1.105 (0.135)	[0.87, 1.40]	1.726* (0.391)	[1.11, 2.69]
**Marital status (ref. Married/in registered partnership)**				
(2) Divorced	1.435 (0.281)	[0.98, 2.11]	1.865 (0.895)	[0.73, 4.78]
(3) Widowed	0.953 (0.331)	[0.48, 1.88]	2.003 (2.328)	[0.20, 19.57]
(4) Single	0.875 (0.149)	[0.63, 1.22]	2.520 (1.385)	[0.86, 7.40]
Information – courses and training	1.157* (0.077)	[1.02, 1.32]	1.030 (0.085)	[0.88, 1.21]
**Occupational areas (ref architecture, surveying, and technical building services)**				
(1) agriculture, forestry, farming, and gardening	0.887 (0.519)	[0.28, 2.79]	0.567 (2.578)	[0.00, 4207.19]
(2) production of raw materials and goods, and manufacturing	0.743 (0.198)	[0.44, 1.25]	1.420 (3.439)	[0.01, 163.77]
(4) construction, natural sciences, geography and informatics	0.627 (0.199)	[0.34, 1.17]	1.225 (3.334)	[0.01, 253.70]
(5) traffic, logistics, safety, and security	0.805 (0.239)	[0.45, 1.44]	2.978 (8.220)	[0.01, 666.15]
(6) commercial services, trading, sales, hotel business and tourism	0.644 (0.195)	[0.36, 1.17]	1.574 (3.873)	[0.01, 195.64]
(7) business organization, accounting, law and administration	0.754 (0.201)	[0.45, 1.27]	2.186 (5.069)	[0.02, 205.63]
(8) health care, the social sector, teaching and education	0.996 (0.271)	[0.58, 1.70]	2.116 (5.180)	[0.02, 256.70]
(9) philology, literature, humanities, social sciences, economics, media, art, culture, and design	0.735 (0.290)	[0.34, 1.59]	1.679 (4.633)	[0.01, 374.86]
**Professional status (ref. Worker)**				
(2) Employee, also employee in public service	1.260 (0.213)	[0.90, 1.75]	3.500 (5.381)	[0.17, 71.25]
(3) Civil servant, also judge, excluding soldiers	1.366 (0.320)	[0.86, 2.16]	3.769 (17.043)	[0.00, 26627.24]
Total working time per week	1.009 (0.005)	[1.00, 1.02]	1.000 (0.010)	[0.98, 1.02]
**Company size (ref. 50 – 99)**				
(1) <5	0.901 (0.270)	[0.50, 1.62]	2.572 (3.201)	[0.22, 29.50]
(2) 5 – 9	0.975 (0.224)	[0.62, 1.53]	1.465 (1.679)	[0.15, 13.85]
(3) 10 – 19	0.914 (0.194)	[0.60, 1.38]	1.201 (1.311)	[0.14, 10.20]
(4) 20 – 49	0.722 (0.137)	[0.50, 1.05]	3.069 (2.800)	[0.51, 18.34]
(6) 100 – 199	1.378 (0.272)	[0.93, 2.03]	0.594 (0.576)	[0.09, 3.97]
(7) 200 – 249	1.081 (0.296)	[0.63, 1.85]	20.897 (42.333)	[0.39, 1107.73]
(8) 250 – 499	1.057 (0.220)	[0.70, 1.59]	3.541 (4.650)	[0.27, 46.44]
(9) 500 – 999	1.124 (0.250)	[0.73, 1.74]	1.494 (1.598)	[0.18, 12.16]
(10) 1,000 – 1,999	1.313 (0.331)	[0.80, 2.15]	2.659 (3.104)	[0.27, 26.23]
(11) ≥2,000	0.852 (0.177)	[0.57, 1.28]	4.073 (4.869)	[0.39, 42.46]
Knowledge-intensive economic sector	1.204 (0.136)	[0.97, 1.50]	0.691 (0.507)	[0.16, 2.92]
Secondary employment	0.829 (0.143)	[0.59, 1.16]	1.501 (0.499)	[0.78, 2.88]
PBC (money)	1.075 (0.057)	[0.97, 1.19]	0.915 (0.060)	[0.80, 1.04]
PBC (time)	0.957 (0.049)	[0.87, 1.06]	0.945 (0.057)	[0.84, 1.06]
Intention*leave of work	−	−	−	−
Intention*PBC (time)	−	−	−	−
**Random**				
Gender (ref. Male)	1.528** (0.196)	[1.19, 1.96]	−	−
Migration background	0.907 (0.121)	[0.70, 1.18]	−	−
Years of education	1.058* (0.027)	[1.01, 1.11]	−	−
Change of company	1.177 (0.242)	[0.79, 1.76]	−	−
Company agreement	1.071 (0.130)	[0.84, 1.36]	−	−
Financing	1.737** (0.299)	[1.24, 2.43]	−	−
Offered financial support	1.357* (0.174)	[1.06, 1.75]	−	−
Planning	1.257 (0.158)	[0.98, 1.61]	−	−
Responsible person	0.867 (0.113)	[0.67, 1.12]	−	−
Leave of work	1.312* (0.174)	[1.01, 1.70]	−	−
Constant^a^	0.022*** (0.015)	[0.01, 0.09]		
Log of the variance	−0.065(0.218)	[–0.49, 0.36]		
Residual standard deviation	0.968 (0.105)	[0.78, 1.20]		
*P* ^b^	0.222 (0.038)	[0.16, 0.30]		

*10 imputations for CVET (outcome). Standard errors are in parentheses.*

*^a^Constant estimates baseline odds.*

*^b^Rho represents the estimated ICC.*

***p < 0.001, **p < 0.01, *p < 0.05.

Regarding Hypothesis 2, we tested whether employer offered *leave of work* as a proxy for ABC has a positive effect on the intention-behavior relation. Following the interpretation for interaction-effects proposed by Rabe-Hesketh and Skrondal (2012), the results displayed in Table 3 reveal that given a *leave of work* within-individual, the estimated conditional odds for CVET participation for an individual without formulating an intention are multiplied by 1.226 and the conditional odds for an individual that formulated an intention are multiplied by 0.996 (=1.226 × 0.812). In terms of percentage change in estimated odds, the conditional odds increase 22,6% [100% (1.226 − 1)] for an individual without an intention and decrease 0.44% [100% (1.226 × 0.812 − 1)] for an individual that formulated an intention. Between-individual, the estimated conditional odds for CVET participation for individuals without an intention are multiplied by 1.226 and the conditional odds for an individual that formulated an intention are multiplied by 1.459 (=1.226 × 1.190). In terms of percentage change in estimated odds, the conditional odds increase 22,60% [100% (1.226 − 1)] for individuals without an intention and increase 45.89% [100% (1.226 × 1.190 − 1)] for individuals that intended to participate. The results reveal, however, that both interactions within- and between-individual are not significant. Hence, we cannot support Hypothesis 2.

**TABLE 3:** Results (Hypothesis 2).

	*N* = 3,788 *n* = 1,894			
	Between-individual		Within-individual	
	OR	CI	OR	CI
Intention	3.587*** (0.875)	[2.22, 5.79]	1.993** (0.513)	[1.20, 3.30]
Age	0.998 (0.007)	[0.98, 1.01]	0.885*** (0.021)	[0.85, 0.93]
**Net household income (ref. 3,000 – 3,999 €)**				
(1) ≤999 €	0.375 (0.342)	[0.06, 2.24]	1.299 (1.282)	[0.19, 8.99]
(2) 1,000 – 1,499 €	1.245 (0.461)	[0.60, 2.57]	1.073 (0.569)	[0.38, 3.03]
(3) 1,500 – 1,999 €	1.292 (0.356)	[0.75, 2.22]	1.125 (0.436)	[0.53, 2.41]
(4) 2,000 – 2,499 €	1.333 (0.315)	[0.84, 2.12]	1.188 (0.328)	[0.69, 2.04]
(5) 2,500 – 2,999 €	0.711 (0.159)	[0.46, 1.10]	0.985 (0.246)	[0.60, 1.61]
(7) 4,000 – 4,999 €	1.080 (0.191)	[0.76, 1.53]	0.697 (0.140)	[0.47, 1.03]
(8) ≥5,000 €	0.829 (0.139)	[0.60, 1.15]	0.622 (0.157)	[0.38, 1.02]
Children	1.104 (0.134)	[0.87, 1.40]	1.730* (0.392)	[1.11, 2.70]
**Marital status (ref. Married/in registered partnership)**				
(2) Divorced	1.435 (0.281)	[0.98, 2.11]	1.866 (0.895)	[0.73, 4.78]
(3) Widowed	0.960 (0.333)	[0.49, 1.89]	1.994 (2.322)	[0.20, 19.57]
(4) Single	0.873 (0.149)	[0.63, 1.22]	2.511 (1.381)	[0.85, 7.38]
Information – courses and training	1.157* (0.076)	[1.02, 1.32]	1.031 (0.085)	[0.88, 1.21]
**Occupational areas (ref architecture, surveying, and technical building services)**				
(1) agriculture, forestry, farming, and gardening	0.896 (0.524)	[0.28, 2.82]	0.645 (2.951)	[0.00, 5069.33]
(2) production of raw materials and goods, and manufacturing	0.745 (0.198)	[0.44, 1.26]	1.402 (3.407)	[0.01, 164.03]
(4) construction, natural sciences, geography and informatics	0.628 (0.199)	[0.34, 1.17]	1.119 (3.056)	[0.01, 236.29]
(5) traffic, logistics, safety, and security	0.801 (0.238)	[0.45, 1.43]	2.914 (8.033)	[0.01, 647.26]
(6) commercial services, trading, sales, hotel business and tourism	0.641 (0.194)	[0.35, 1.16]	1.536 (3.788)	[0.01, 192.94]
(7) business organization, accounting, law and administration	0.754 (0.201)	[0.45, 1.27]	2.081 (4.837)	[0.02, 198.12]
(8) health care, the social sector, teaching and education	0.999 (0.272)	[0.59, 1.70]	2.024 (4.968)	[0.02, 248.69]
(9) philology, literature, humanities, social sciences, economics, media, art, culture, and design	0.737 (0.290)	[0.34, 1.59]	1.547 (4.282)	[0.01, 351.01]
**Professional status (ref. Worker)**				
(2) Employee, also employee in public service	1.268 (0.214)	[0.91, 1.77]	3.466 (5.321)	[0.17, 70.25]
(3) Civil servant, also judge, excluding soldiers	1.377 (0.323)	[0.87, 2.18]	3.790 (17.216)	[0.00, 27874.94]
Total working time per week	1.009 (0.005)	[1.00, 1.02]	1.000 (0.010)	[0.98, 1.02]
**Company size (ref. 50 – 99)**				
(1) <5	0.895 (0.268)	[0.50, 1.61]	2.629 (3.272)	[0.23, 30.16]
(2) 5 – 9	0.977 (0.225)	[0.62, 1.53]	1.496 (1.714)	[0.16, 14.13]
(3) 10 – 19	0.916 (0.194)	[0.60, 1.39]	1.207 (1.318)	[0.14, 10.26]
(4) 20 – 49	0.725 (0.137)	[0.50, 1.05]	3.141 (2.869)	[0.52, 18.82]
(6) 100 – 199	1.383 (0.273)	[0.94, 2.04]	0.598 (0.580)	[0.09, 4.01]
(7) 200 – 249	1.083 (0.296)	[0.63, 1.85]	20.906 (42.457)	[0.39, 1119.21]
(8) 250 – 499	1.061 (0.221)	[0.70, 1.60]	3.548 (4.664)	[0.27, 46.67]
(9) 500 – 999	1.126 (0.251)	[0.73, 1.74]	1.527 (1.638)	[0.19, 12.52]
(10) 1,000 – 1,999	1.308 (0.330)	[0.80, 2.14]	2.711 (3.171)	[0.27, 26.86]
(11) ≥2,000	0.853 (0.177)	[0.57, 1.28]	4.136 (4.952)	[0.40, 43.28]
Knowledge-intensive economic sector	1.206 (0.136)	[0.97, 1.50]	0.693 (0.510)	[0.16, 2.93]
Secondary employment	0.833 (0.144)	[0.59, 1.17]	1.494 (0.497)	[0.78, 2.87]
PBC (money)	1.074 (0.057)	[0.97, 1.19]	0.914 (0.060)	[0.80, 1.04]
PBC (time)	0.957 (0.049)	[0.87, 1.06]	0.947 (0.057)	[0.84, 1.07]
Intention*leave of work	1.190 (0.328)	[0.69, 2.04]	0.812 (0.243)	[0.45, 1.46]
Intention*PBC (time)	−	−	−	−
**Random**				
Gender (ref. Male)	1.533** (0.197)	[1.19, 1.97]	−	−
Migration background	0.906 (0.121)	[0.70, 1.18]	−	−
Years of education	1.058* (0.027)	[1.01, 1.11]	−	−
Change of company	1.176 (0.242)	[0.79, 1.76]	−	−
Company agreement	1.067 (0.129)	[0.84, 1.35]	−	−
Financing	1.757** (0.303)	[1.25, 2.46]	−	−
Offered financial support	1.363* (0.175)	[1.06, 1.75]	−	−
Planning	1.258 (0.158)	[0.98, 1.61]	−	−
Responsible person	0.867 (0.113)	[0.67, 1.12]	−	−
Leave of work	1.226 (0.212)	[0.87, 1.72]	−	−
Constant^a^	0.022*** (0.015)	[0.01, 0.09]		
Log of the variance	−0.065(0.218)	[–0.49, 0.36]		
Residual standard deviation	0.968 (0.106)	[0.78, 1.20]		
ρ^b^	0.222 (0.038)	[0.16, 0.30]		

*10 imputations for CVET (outcome). Standard errors are in parentheses.*

*^a^Constant estimates baseline odds.*

*^b^Rho represents the estimated ICC.*

***p < 0.001, **p < 0.01, *p < 0.05.

Regarding Hypothesis 3, we tested whether the degree of *perceived behavioral control* regarding the availability of temporal resources to participate in CVET moderates the intention-behavior relation (Table 4). Following Rabe-Hesketh and Skrondal (2012), the results reveal that given a one-unit increase regarding the perceived availability of temporal resources within-individual, the estimated conditional odds for CVET participation for an individual without formulating an intention are multiplied by 0.960 and the conditional odds for an individual that formulated an intention are multiplied by 0.927 (=0.960 × 0.966). In terms of percentage change in estimated odds, the conditional odds decrease 4% [100% (0.960 − 1)] for an individual without an intention and decrease 7.26% [100% (0.960 × 0.966 − 1)] for an individual that formulated an intention. Between-individual, the estimated conditional odds for CVET participation given a one-unit increase regarding the perceived availability of temporal resources for individuals without an intention are multiplied by 0.876 (*p* < 0.05) for an individual that formulated an intention are multiplied by 1.093 (=0.876 × 1.248). In terms of percentage change in estimated odds, the conditional odds decrease 12.40% [100% (0.876 − 1)] for individuals without an intention and increase 9.32% [100% (0.876 × 1.248 − 1)] for individuals that intended to participate. The results reveal, however, that only the interaction effect between-individual is significant (OR 1.248, *p* < 0.05). However, despite this significant interaction effect, we cannot interpret this effect in terms of causal inference “because it is confounded with the level 2 error” (Schunck, 2013, p. 69). Drawing causal inferences requires looking at within individual changes over time (Allison, 2009). Therefore, we reject Hypothesis 3.

**TABLE 4:** Results (Hypothesis 3).

	*N* = 3,788 *n* = 1,894			
	Between-individual		Within-individual	
	OR	CI	OR	CI
Intention	1.928 (0.725)	[0.92, 4.03]	1.918 (0.676)	[0.96, 3.83]
Age	0.998 (0.007)	[0.98, 1.01]	0.885*** (0.021)	[0.85, 0.93]
**Net household income(ref. 3,000 – 3,999 €)**				
(1)≤ 999 €	0.362 (0.331)	[0.06, 2.18]	1.337 (1.321)	[0.19, 9.28]
(2) 1,000 – 1,499 €	1.211 (0.449)	[0.59, 2.51]	1.074 (0.570)	[0.38, 3.04]
(3) 1,500 – 1,999 €	1.296 (0.357)	[0.76, 2.23]	1.122 (0.435)	[0.52, 2.40]
(4) 2,000 – 2,499 €	1.329 (0.314)	[0.84, 2.11]	1.187 (0.328)	[0.69, 2.04]
(5) 2,500 – 2,999 €	0.700 (0.157)	[0.45, 1.09]	0.980 (0.245)	[0.60, 1.60]
(7) 4,000 – 4,999 €	1.059 (0.188)	[0.75, 1.50]	0.696 (0.140)	[0.47, 1.03]
(8) ≥5,000 €	0.829 (0.139)	[0.60, 1.15]	0.623 (0.157)	[0.38, 1.02]
Children	1.117 (0.136)	[0.88, 1.42]	1.728* (0.392)	[1.11, 2.69]
**Marital status (ref. Married/in registered partnership)**				
(2) Divorced	1.444 (0.283)	[0.98, 2.12]	1.858 (0.894)	[0.72, 4.78]
(3) Widowed	0.968 (0.336)	[0.49, 1.91]	1.993 (2.314)	[0.20, 19.44]
(4) Single	0.882 (0.150)	[0.63, 1.23]	2.496 (1.377)	[0.85, 7.36]
Information – courses and training	1.155* (0.076)	[1.01, 1.32]	1.031 (0.085)	[0.88, 1.21]
**Occupational areas (ref architecture, surveying, and technical building services)**				
(1) agriculture, forestry, farming, and gardening	0.924 (0.541)	[0.29, 2.91]	0.577 (2.614)	[0.00, 4152.94]
(2) production of raw materials and goods, and manufacturing	0.750 (0.200)	[0.45, 1.27]	1.402 (3.432)	[0.01, 169.81]
(4) construction, natural sciences, geography and informatics	0.635 (0.201)	[0.34, 1.18]	1.218 (3.345)	[0.01, 265.38]
(5) traffic, logistics, safety, and security	0.806 (0.239)	[0.45, 1.44]	2.979 (8.348)	[01, 723.45]
(6) commercial services, trading, sales, hotel business and tourism	0.650 (0.197)	[0.36, 1.18]	1.539 (3.828)	[01, 201.52]
(7) business organization, accounting, law and administration	0.768 (0.205)	[0.46, 1.29]	2.178 (5.108)	[0.02, 216.10]
(8) health care, the social sector, teaching and education	1.022 (0.279)	[0.60, 1.74]	2.092 (5.172)	[0.02, 266.11]
(9) philology, literature, humanities, social sciences, economics, media, art, culture, and design	0.777 (0.306)	[0.36, 1.68]	1.660 (4.613)	[0.01, 385.40]
**Professional status (ref. Worker)**				
(2) Employee, also employee in public service	1.255 (0.212)	[0.90, 1.75]	3.504 (5.403)	[0.17, 71.99]
(3) Civil servant, also judge, excluding soldiers	1.384 (0.324)	[0.87, 2.19]	3.796 (17.249)	[0.00, 27984.23]
Total working time per week	1.009 (0.005)	[1.00, 1.02]	1.001 (0.010)	[0.98, 1.02]
**Company size (ref. 50 – 99)**				
(1) <5	0.886 (0.265)	[0.49, 1.59]	2.574 (3.200)	[0.23, 29.44]
(2) 5 – 9	0.970 (0.223)	[0.62, 1.52]	1.442 (1.652)	[0.15, 13.61]
(3) 10 – 19	0.907 (0.192)	[0.60, 1.37]	1.196 (1.300)	[0.14, 10.07]
(4) 20 – 49	0.717 (0.136)	[0.50, 1.04]	3.054 (2.788)	[0.51, 18.28]
(6) 100 – 199	1.369 (0.270)	[0.93, 2.01]	0.599 (0.577)	[0.09, 3.96]
(7) 200 – 249	1.082 (0.296)	[0.63, 1.85]	20.783 (42.083)	[0.39, 1099.63]
(8) 250 – 499	1.038 (0.217)	[0.69, 1.56]	3.501 (4.579)	[0.27, 45.45]
(9) 500 – 999	1.118 (0.249)	[0.72, 1.73]	1.461 (1.562)	[0.18, 11.89]
(10) 1,000 – 1,999	1.296 (0.327)	[0.79, 2.13]	2.625 (3.058)	[0.27, 25.75]
(11) ≥2,000	0.857 (0.178)	[0.57, 1.29]	4.079 (4.863)	[0.39, 42.26]
Knowledge-intensive economic sector	1.210 (0.136)	[0.97, 1.51]	0.693 (0.507)	[0.17, 2.91]
Secondary employment	0.829 (0.143)	[0.59, 1.16]	1.495 (0.497)	[0.78, 2.87]
PBC (money)	1.074 (0.057)	[0.97, 1.19]	0.914 (0.060)	[0.80, 1.04]
PBC (time)	0.876* (0.058)	[0.77, 1.00]	0.960 (0.072)	[0.83, 1.11]
Intention*leave of work	−	−	−	−
Intention*PBC (time)	1.248* (0.130)	[1.02, 1.53]	0.966 (0.096)	[0.80, 1.17]
**Random**				
Gender (ref. Male)	1.531** (0.196)	[1.19, 1.97]	−	−
Migration background	0.916 (0.122)	[0.71, 1.19]	−	−
Years of education	1.060* (0.027)	[1.01, 1.12]	−	−
Change of company	1.187 (0.244)	[0.79, 1.78]	−	−
Company agreement	1.080 (0.131)	[0.85, 1.37]	−	−
Financing	1.733** (0.298)	[1.24, 2.43]	−	−
Offered financial support	1.352* (0.173)	[1.05, 1.74]	−	−
Planning	1.251 (0.157)	[0.98, 1.60]	−	−
Responsible person	0.863 (0.112)	[0.67, 1.11]	−	−
Leave of work	1.311* (0.173)	[1.01, 1.70]	−	−
Constant^a^	0.028*** (0.019)	[0.01, 0.11]		
Log of the variance	−0.074(0.219)	[–0.50, 0.36]		
Residual standard deviation	0.964 (0.105)	[0.78, 1.19]		
ρ^b^	0.220 (0.038)	[0.16, 0.30]		

*10 imputations for CVET (outcome). Standard errors are in parentheses.*

*^a^Constant estimates baseline odds.*

*^b^Rho represents the estimated ICC.*

***p < 0.001, **p < 0.01, *p < 0.05.

Regarding Hypothesis 4, we tested whether PBC can only be used as a proxy for ABC in moderating the intention-behavior relation. To test this Hypothesis, we estimated the model from Hypothesis 3 for two subgroups, distinguishing individuals who were not offered a *leave of work* (Table 5) and individuals who were offered a *leave of work* for CVET participation (Table 6). Regarding the subgroup of individuals that were not offered a *leave of work*, the results displayed in Table 5 reveal no significant interaction effects of an intention and the degree of perceived behavioral control on CVET participation, neither within-individual (OR = 0.944), nor between-individual (OR = 1.514).

**TABLE 5:** Results (Hypothesis 4 – subsample of individuals who were not offered a leave of work).

	*N = 1,438 n = 719*			
	Between-individual		Within-individual	
	OR	CI	OR	CI
Intention	0.835 (0.676)	[0.17, 4.08]	2.430 (1.661)	[0.64, 9.28]
Age	0.975 (0.014)	[0.95, 1.00]	0.903* (0.041)	[0.83, 0.99]
**Net household income (ref. 3,000 – 3,999 €)**				
(1) ≤999 €	4.50e-09 (0.000)	[–1.27, 0.]	1.27e+08 (1.84e+12)	[0, 0.0]
(2) 1,000 – 1,499 €	1.305 (0.906)	[0.33, 5.09]	0.711 (0.664)	[0.11, 4.45]
(3) 1,500 – 1,999 €	1.553 (0.814)	[0.56, 4.34]	0.244* (0.170)	[0.06, 0.95]
(4) 2,000 – 2,499 €	0.995 (0.438)	[0.42, 2.36]	1.123 (0.536)	[0.44, 2.86]
(5) 2,500 – 2,999 €	1.181 (0.500)	[0.51, 2.71]	0.676 (0.301)	[0.28, 1.62]
(7) 4,000 – 4,999 €	1.068 (0.402)	[0.51, 2.23]	0.829 (0.333)	[0.38, 1.82]
(8) ≥5,000 €	0.616 (0.235)	[0.29, 1.30]	1.313 (0.700)	[0.46, 3.74]
Children	1.261 (0.309)	[0.78, 2.04]	2.281 (1.088)	[0.90, 5.81]
**Marital status (ref. Married/in registered partnership)**				
(2) Divorced	1.091 (0.434)	[0.50, 2.38]	2.639 (2.044)	[0.58, 12.04]
(3) Widowed	0.905 (0.542)	[0.28, 2.93]	3.071 (7.161)	[0.03, 296.56]
(4) Single	0.844 (0.297)	[0.42, 1.68]	36.154* (50.099)	[2.38, 549.41]
Information – courses and training	1.284 (0.164)	[1.00, 1.65]	1.204 (0.185)	[0.89, 1.63]
**Occupational areas (ref architecture, surveying, and technical building services)**				
(1) agriculture, forestry, farming, and gardening	0.843 (0.895)	[0.11, 6.75]	24.704 (164.96)	[0.00, 1.19e+07]
(2) production of raw materials and goods, and manufacturing	0.923 (0.470)	[0.34, 2.50]	0.531 (2.451)	[0.00, 4474.01]
(4) construction, natural sciences, geography and informatics	1.017 (0.700)	[0.26, 3.92]	0.021 (0.140)	[3.86e-08, 11192.51]
(5) traffic, logistics, safety, and security	0.952 (0.519)	[0.33, 2.77]	0.141 (0.690)	[9.35e-06, 2116.93]
(6) commercial services, trading, sales, hotel business and tourism	0.677 (0.390)	[0.22, 2.09]	1.159 (5.949)	[0.00, 27044.20]
(7) business organization, accounting, law and administration	1.021 (0.558)	[0.35, 2.98]	0.160 (0.858)	[4.28e-06, 5952.69]
(8) health care, the social sector, teaching and education	1.844 (1.052)	[0.60, 5.64]	0.058 (0.343)	[5.14e-07, 6492.09]
(9) philology, literature, humanities, social sciences, economics, media, art, culture, and design	0.635 (0.488)	[0.14, 2.87]	0.106 (0.746)	[1.06e-07, 106047.40]
**Professional status (ref. Worker)**				
(2) Employee, also employee in public service	1.025 (0.299)	[0.58, 1.82]	0.243 (0.650)	[0.00, 45.94]
(3) Civil servant, also judge, excluding soldiers	0.815 (0.445)	[0.28, 2.38]	1.000 (omitted)	[,]
Total working time per week	1.029** (0.011)	[1.01, 1.05]	0.994 (0.018)	[0.96, 1.03]
**Company size (ref. 50 – 99)**				
(1) <5	0.727 (0.371)	[0.27, 1.98]	2.751 (7.144)	[0.02, 447.07]
(2) 5 – 9	0.536 (0.249)	[0.22, 1.33]	3.023 (7.617)	[0.02, 423.25]
(3) 10 – 19	0.643 (0.273)	[0.28, 1.48]	3.669 (8.715)	[0.03, 387.80]
(4) 20 – 49	0.716 (0.258)	[0.35, 1.45]	2.217 (3.371)	[0.11, 43.66]
(6) 100 – 199	0.981 (0.378)	[0.46, 2.09]	1.315 (2.137)	[0.05, 31.78]
(7) 200 – 249	0.962 (0.532)	[0.33, 2.84]	1.000 (omitted)	−
(8) 250 – 499	0.757 (0.309)	[0.34, 1.68]	418.242 (1513.437)	[0.35, 503347.30]
(9) 500 – 999	0.723 (0.357)	[0.27, 1.90]	0.262 (0.871)	[0.00, 175.36]
(10) 1,000 – 1,999	1.070 (0.623)	[0.34, 3.35]	44.717 (133.103)	[0.13, 15286.19]
(11) ≥2,000	1.202 (0.562)	[0.48, 3.00]	2.387 (6.550)	[0.01, 517.38]
Knowledge-intensive economic sector	1.348 (0.318)	[0.85, 2.14]	0.631 (1.046)	[0.02, 16.32]
Secondary employment	1.212 (0.445)	[0.59, 2.49]	1.351 (0.950)	[0.34, 5.36]
PBC (money)	1.004 (0.104)	[0.82, 1.23]	0.977 (0.121)	[0.77, 1.25]
PBC (time)	0.916 (0.109)	[0.73, 1.16]	1.015 (0.135)	[0.78, 1.32]
Intention*leave of work	−	−	−	−
Intention*PBC (time)	1.514 (0.358)	[0.95, 2.41]	0.944 (0.188)	[0.64, 1.39]
**Random**				
Gender (ref. Male)	1.847* (0.487)	[1.10, 3.09]		
Migration background	0.628 (0.163)	[0.38, 1.04]		
Years of education	1.181** (0.065)	[1.06, 1.31]		
Change of company	0.675 (0.305)	[0.28, 1.64]		
Company agreement	0.816 (0.206)	[0.50, 1.34]		
Financing	1.287 (0.328)	[0.78, 2.12]		
Offered financial support	1.253 (0.312)	[0.77, 2.04]		
Planning	1.999** (0.520)	[1.20, 3.33]		
Responsible person	0.891 (0.229)	[0.54, 1.48]		
Leave of work	−	−		
Constant^a^	0.008*** (0.011)	[0.00, 0.12]		
Log of the variance	0.538 (0.309)	[–0.07, 1.14]		
Residual standard deviation	1.309 (0.202)	[0.97, 1.77]		
ρ^b^	0.342 (0.070)	[0.22, 0.49]		

*10 imputations for CVET (outcome). Standard errors are in parentheses.*

*^a^Constant estimates baseline odds.*

*^b^Rho represents the estimated ICC.*

***p < 0.001, **p < 0.01, *p < 0.05.

**TABLE 6:** Results (Hypothesis 4 – subsample of individuals who were offered a leave of work).

	*N = 2,350 n = 1,175*			
	Between-individual		Within-individual	
	OR	CI	OR	CI
Intention	2.751** (1.252)	[1.13, 6.72]	1.459 (0.630)	[0.63, 3.40]
Age	1.006 (0.008)	[0.99, 1.02]	0.869*** (0.025)	[0.82, 0.92]
**Net household income (ref. 3,000 – 3,999 €)**				
(1) ≤999 €	0.979 (1.347)	[0.07, 14.52]	0.181 (0.279)	[0.01, 3.72]
(2) 1,000 – 1,499 €	1.197 (0.559)	[0.48, 2.99]	1.570 (1.098)	[0.40, 6.18]
(3) 1,500 – 1,999 €	1.242 (0.434)	[0.63, 2.46]	3.343* (1.737)	[1.21, 9.26]
(4) 2,000 – 2,499 €	1.711 (0.525)	[0.94, 3.12]	1.245 (0.453)	[0.61, 2.54]
(5) 2,500 – 2,999 €	0.524* (0.145)	[0.30, 0.90]	1.158 (0.350)	[0.64, 2.09]
(7) 4,000 – 4,999 €	1.090 (0.232)	[0.72, 1.65]	0.688 (0.165)	[0.43, 1.10]
(8) ≥5,000 €	0.950 (0.182)	[0.65, 1.38]	0.535* (0.157)	[0.30, 0.95]
Children	1.062 (0.152)	[0.80, 1.41]	1.628 (0.434)	[0.97, 2.74]
**Marital status (ref. Married/in registered partnership)**				
(2) Divorced	1.733* (0.416)	[1.08, 2.78]	1.777 (1.141)	[0.50, 6.26]
(3) Widowed	1.010 (0.481)	[0.40, 2.57]	1.552 (2.123)	[0.11, 22.76]
(4) Single	0.863 (0.171)	[0.59, 1.27]	1.427 (0.903)	[0.41, 4.94]
Information – courses and training	1.087 (0.087)	[0.93, 1.27]	0.945 (0.097)	[0.77, 1.15]
**Occupational areas (ref architecture, surveying, and technical building services)**				
(1) agriculture, forestry, farming, and gardening	0.655 (0.481)	[0.16, 2.77]	1 (omitted)	[,]
(2) production of raw materials and goods, and manufacturing	0.630 (0.209)	[0.33, 1.21]	4.564 (18.108)	[0.00, 10877.46]
(4) construction, natural sciences, geography and informatics	0.518 (0.189)	[0.25, 1.06]	0.582 (2.291)	[0.00, 1309.25]
(5) traffic, logistics, safety, and security	0.773 (0.298)	[0.36, 1.65]	1.189 (6.571)	[0.00, 60449.68]
(6) commercial services, trading, sales, hotel business and tourism	0.613 (0.231)	[0.29, 1.28]	0.507 (1.851)	[0.00, 646.75]
(7) business organization, accounting, law and administration	0.627 (0.198)	[0.34, 1.16]	1.700 (5.369)	[0.00, 830.44]
(8) health care, the social sector, teaching and education	0.789 (0.252)	[0.42, 1.48]	1.050 (3.710)	[0.00, 1066.70]
(9) philology, literature, humanities, social sciences, economics, media, art, culture, and design	0.852 (0.416)	[0.33, 2.22]	0.594 (2.203)	[0.00, 851.15]
**Professional status (ref. Worker)**				
(2) Employee, also employee in public service	1.356 (0.306)	[0.87, 2.11]	339.55 (1171.62)	[0.39, 293799.20]
(3) Civil servant, also judge, excluding soldiers	1.495 (0.425)	[0.86, 2.61]	467.80 (2705.81)	[0.01, 3.92e+07]
Total working time per week	1.001 (0.006)	[0.99, 1.01]	1.001 (0.012)	[0.98, 1.03]
**Company size (ref. 50 – 99)**				
(1) <5	1.009 (0.404)	[0.46, 2.21]	5.796 (10.638)	[0.16, 211.72]
(2) 5 – 9	1.197 (0.327)	[0.70, 2.04]	2.630 (4.084)	[0.13, 55.16]
(3) 10 – 19	0.971 (0.245)	[0.59, 1.59]	0.545 (0.870)	[0.02, 12.46]
(4) 20 – 49	0.646 (0.147)	[0.41, 1.01]	8.848 (11.436)	[0.70, 111.43]
(6) 100 – 199	1.529 (0.362)	[0.96, 2.43]	0.389 (0.541)	[0.03, 5.94]
(7) 200 – 249	1.160 (0.377)	[0.61, 2.19]	8.544 (18.838)	[0.11, 643.20]
(8) 250 – 499	1.178 (0.295)	[0.72, 1.92]	1.272 (2.176)	[0.04, 36.41]
(9) 500 – 999	1.222 (0.313)	[0.74, 2.02]	1.548 (2.069)	[0.11, 21.28]
(10) 1,000 – 1,999	1.345 (0.380)	[0.77, 2.34]	1.545 (2.477)	[0.07, 35.87]
(11) ≥2,000	0.818 (0.195)	[0.51, 1.31]	6.918 (11.212)	[0.29, 166.18]
Knowledge-intensive economic sector	1.150 (0.145)	[0.90, 1.47]	0.922 (0.847)	[0.15, 5.60]
Secondary employment	0.744 (0.149)	[0.50, 1.10]	1.439 (0.575)	[0.66, 3.15]
PBC (money)	1.118 (0.072)	[0.98, 1.27]	0.867 (0.070)	[0.74, 1.02]
PBC (time)	0.884 (0.075)	[0.75, 1.04]	0.912 (0.087)	[0.76, 1.10]
Intention*leave of work	−	−	−	−
Intention*PBC (time)	1.156 (0.143)	[0.91, 1.47]	1.023 (0.123)	[0.81, 1.29]
**Random**				
Gender (ref. Male)	1.444* (0.212)	[1.08, 1.93]		
Migration background	1.022 (0.168)	[0.74, 1.41]		
Years of education	1.021 (0.031)	[0.96, 1.08]		
Change of company	1.716* (0.433)	[1.05, 2.81]		
Company agreement	1.147 (0.161)	[0.87, 1.51]		
Financing	2.051* (0.665)	[1.09, 3.87]		
Offered financial support	1.323 (0.211)	[0.97, 1.81]		
Planning	1.020 (0.152)	[0.76, 1.37]		
Responsible person	0.837 (0.133)	[0.61, 1.14]		
Leave of work	−	−		
Constant^a^	0.063** (0.057)	[0.01, 0.37]		
Log of the variance	−0.431(0.338)	[–1.09, 0.23]		
Residual standard deviation	0.806 (0.136)	[0.58, 1.12]		
ρ^b^	0.165 (0.047)	[0.09, 0.28]		

*10 imputations for CVET (outcome). Standard errors are in parentheses.*

*^a^Constant estimates baseline odds.*

*^b^Rho represents the estimated ICC.*

***p < 0.001, **p < 0.01, *p < 0.05.

In addition, the results for the individuals that were offered a *leave of work* (Table 6) also reveal no significant interaction effects, neither within-individual (OR = 1.023), nor between-individual (OR = 1.156). Because of the non-significant interaction effects, we decided to not give full interpretations of percentage changes in estimated odds at this point. Consequently, we cannot confirm Hypothesis 4.

## Discussion

Lack of time is one of the most common reasons adults give for not participating in educational activities. Yet few studies aiming at causal inferences tested the relationship of the availability of temporal resources with participation behavior. In view of the importance of time for participation, the present study puts effort in researching the impact of employer offered *leave of work* on CVET participation. Overall, the results both confirm findings from current research regarding participation as an intentional behavior and its determinants and contribute to new knowledge on how CVET participation is affected by time. The present study provides four main conclusions:

First, participation in CVET is an intentional behavior (Hypothesis 1). The results support evidence from current research (e.g., Kyndt and Baert, 2013) that the intention to participate is related to participation in CVET.

Second, the results displayed in Table 2 reveal that support for CVET by the employer significantly increases the conditional odds of CVET participation between-individual. The results support current evidence on the importance of organizational support for CVET (e.g., Hurtz and Williams, 2009; Kaufmann and Widany, 2013; Lischewski et al., 2020). Based on the results, financing or providing classes or training courses as well as offering financial support increase the conditional odds by, respectively, 73,68% (financing; *p* < 0.01) and 35,73% (offered financial support; *p* < 0.05). Employer offered *leave of work* increases the conditional odds by 31,23% (*p* < 0.05). The conditional odds ratios for education planning and company agreement are positive but not significant. The conditional odds ratio for responsible person is negative and not significant. Based on these results, the employer can support CVET participation significantly by providing financial contributions and by offering timeframes for CVET. However, it is important to mention that the conditional odds ratios for time-constant predictors such as *leave of work* on the company-level “do *not* control for unmeasured predictors” (Allison, 2009, p. 41) and thus cannot be interpreted in terms of causal inferences.

Third, despite individuals offered a *leave of work* have significantly higher rates of CVET participation, we found no significant interaction effect of an intention to participate and *leave of work* on CVET participation (Hypothesis 2). Thus, we cannot support the hypothesis that *leave of work* is a moderator of the intention-behavior relation. However, *leave of work* impacts both intention and participation as a confounder. Therefore, *leave of work* not only affects those individuals that intend to participate but also affects those who have not formulated an intention but participate in CVET anyway. Accordingly, we found the non-significant effect that the conditional odds of CVET participation within-individual increase by 22,6% for an individual without an intention to participate (Table 3). A possible explanation for this finding is that *leave of work* is also associated with an expectation on the part of the employer to participate, even if no intention has been formed on the part of the employee at the time of the interview. In this case, CVET participation is not only the result of an individual decision-making process but also of an employer-related decision and thus an external selection (Kaufmann and Widany, 2013). Therefore, *leave of work* may not only be a proxy for ABC, but may also represent a social norm on the part of the employer and thus influence the formation of an intention.

Forth, we found mixed results when testing the hypothesized effects of PBC on the intention-behavior relation. On the one hand, the results provide evidence that within-individual, the degree of perceived behavioral control regarding the availability of temporal resources to participate in CVET does neither moderate the intention-behavior relation (Hypothesis 3) nor is it a proxy for ABC (Hypothesis 4). One the other hand, we found a significant interaction effect between-individual (Table 4; OR 1.248, *p* < 0.05). This indicates that a between-individual increase of the degree of perceived behavioral control increases the conditional odds of CVET participation significantly. This finding supports the assumption in current literature that someone “who is only slightly interested in learning will be likely to think that he or she has no time for it (other interests take precedence) and make no room for it, while someone who is very interested will probably make more effort to find a solution to practical barriers (Baert et al., 2006, p. 97).

In addition, our study provides further interesting results. Here, we refer to the results displayed in Table 2. Within-individual, we find that increases in age are associated with significant decreases of the conditional odds of CVET participation (OR 0.886; *p* < 0.001). Overall, the influence of age on CVET participation remains ambiguous in current literature (Lischewski et al., 2020). However, it can be assumed that in line with human capital theory the payoff of investments in CVET decreases with age (Becker, 2019). Regarding household characteristics, we find that a change from having no children in the household to having children increases the conditional odds by 72.60% (*p* < 0.05). This is a rather surprising result because current academia assumes that children in the household reduce the availability of temporary and monetary resources and thus decrease the probability of participation (e.g., Lischewski et al., 2020). Between-individual, we find that a one-unit increase regarding the information about CVET programs and courses increases the conditional odds of CVET significantly by 15.70% (OR = 1.157, *p* < 0.05). Furthermore, we find that women are 1.559 (*p* < 0.01) times more likely to participate in CVET than men.

## Limitations

Although this is one of the first longitudinal studies that aims at causal inferences when investigating the impact of time on CVET participation, some limitations must be considered.

Regarding the behavioral intention, applying the TPB usually involves asking respondents to rate “how strongly they intend to perform the behavior” (Fishbein and Ajzen, 2010, p. 43). The NEPS does not provide such a measurement of the strength of an intention, but an operationalization as a dichotomous variable. This leaves the possibility open, that some of the surveyed respondents had a stronger intention to participate than others. However, the results (Table 2) revealed that an intention to participate significantly predicts CVET participation both within- and between-individual.

By employing an imputation method for CVET participation, we created a dummy variable for every respondent and year that identifies whether a respondent participated in at least one course of CVET. Accordingly, our analyses provide no information regarding the segments of CVET (BMBF, 2017, 2019). However, the aim of our study was to analyze the conditions of implementing an intention to participate in CVET. Based on the universal rationale of the intention-behavior relation as theorized by the TPB, the estimation results of Hypothesis 1 provide generic knowledge that CVET participation is an intentional behavior.

We used *leave of work* as a proxy for ABC and investigated its theorized moderating role on the intention-behavior relation. However, based on the NEPS data, we were only able to use *leave of work* as an inter-individual variable. Thus, no effects of a change of employer offered *leave of work* on CVET participation within-individual were estimated.

With the concept of PBC, the TPB considers that the individual perception of the availability of information, abilities, opportunities, and resources that are required to perform a behavior impacts the intention-behavior relation. However, the NEPS only provides information on the perception of the availability of temporal and monetary recourses as well as of knowledge regarding CVET courses and programs. This leaves the possibility open, that the intention-behavior relation might be affected by the perceived availability of opportunities. In this regard, scholars emphasize that it “is important to recognize that individuals’ choices to (not) participate are also influenced by the education and training opportunities available to them” (Boeren, 2017, p. 163). This, for example includes the temporal availability of, and accessibility to a supply of course offerings by educational institutions (Rüter and Martin, 2021).

Furthermore, based on the NEPS data, we were not able to investigate the proximal determinants of intentions. The revised model of the TBP conceptualizes PBC as a moderator of the influences of attitude and social norms on an intention (Ajzen, 2020). Accordingly, formulating an intention includes a perception of a sufficiently high degree of behavioral control about the availability of temporal resources. Although this assumption seems reasonable, we could not investigate the proximal determinants of an intention.

On the employer-level, *leave of work* is a measure to encourage and support CVET participation of employees. Based on our research design, we are not able to formulate any statements regarding the decision on part of the employer to offer a *leave of work*. In addition, no cross-level interactions between employees and employer were investigated. Such a research design would require a nested structure of employees nested in companies. However, “factors that determine who does, and does not, receive the opportunity to participate in adult education are key issues” (Saar and Räis, 2017, p. 531). This refers both to the participation itself as well as its theoretical and empirical explanation. Unfortunately, the question about the conditions under which CVET is supported by the employer remains unanswered in our study. In this regard, the descriptive statistics of the two subsamples of Hypothesis 4 (Table 1) reveal differences between individuals who were offered a *leave of work* by their employer and those without such a support.

## Conclusion

The TPB provides a heuristic framework for our study. The theory allowed us to explain CVET participation as a process from formulating an intention to actual participation. In addition, the TPB allows the integration of individual and contextual factors (e.g., the work environment) in explaining individual behavior and the underpinning decision-making process. We tested the theorized interaction effects of both PBC and ABC on the intention-behavior relation with the focus on how the perceived and actual availability of temporal resources affects CVET participation. We used employer offered *leave of work* as a proxy for ABC. However, we found no evidence in support of this theorized interaction. What are the implications of the results for the development of the TPB and the investigation of individual participation behavior? According to the revised model of the TPB (Ajzen, 2012, 2020), any behavior is a function of a behavioral intention and actual behavior control. However, we found no significant support for this hypothesis regarding the availability of temporal recourses in terms of *leave of work*. The intention-behavior relation is part of a continuous development of the theory of planned behavior (Bosnjak et al., 2020; La Barbera and Ajzen, 2021). In this regard, the conditions under which an intention leads to the performance of a behavior are of particular relevance. Regarding the implementation of intentions, Opp (2019) recently criticized, that the question of why individuals choose to perform a behavior remains unanswered by the current theoretical model proposed by the TPB. To address this issue, researcher currently argue that it could be interesting and fruitful to complement the TPB with elements of other behavioral theories. Recently, Opp (2019) suggested integrating the TPB with the value-expectancy theory (VET). VET explains behavior in the way that a behavior is performed in dependence of perceived behavioral alternatives. “The alternative chosen is a function of the *perceived behavioral consequences or outcomes* (…), their *subjective probabilities* (…) and their *utilities* (…)” (Opp, 2019, p. 74). Complementing TPB and VET includes adding goals, subjective utility maximization and behavioral consequences (VET) as well as the performance of a behavior as a result of an intention (TPB) into an integrated model. In the proposed model, goals in a first step are integrated as a major determinant of behavior. Thus, an individual performs a given behavior to reach a certain goal. A goal impacts the formulation of an intention which then predicts the behavior. A second step is the inclusion of the assumption of subjective utility maximization. Opp (2019) argues that an intention originates for a behavior that has the highest subjective expected utility. Accordingly, in the context of adult learning, “a learning intention can be defined as a readiness or even a plan to undertake a concrete action in order to neutralize the experienced discrepancy, and to reach a desired situation by means of training and education” (Kyndt et al., 2011, p. 215). This includes a perception of available resources, knowledge, opportunities, etc. In addition, the integrated model approaches ABC not as moderator of the intention-behavior relation, but rather as a “scope condition” (Opp, 2019, p. 91) to perform the behavior. The intention-behavior relation is theorized to be moderated by PBC. Third, relevant behavioral consequences of the behavioral performance that include positive or negative utilities are added as an independent predictor of behavior.

How the integration of goals, behavioral consequences, and subjective utility maximation could improve the investigation and explanation of the intention-behavior relation is an empirical question that should be addressed in future research. A first research question in this regard could for example be about the goals associated with the intention to participate in CVET and whether these goals could be achieved by participating in CVET.

## Data Availability Statement

Publicly available datasets were analyzed in this study. This manuscript uses data from the German National Educational Panel Study (NEPS): Starting Cohort Adults, doi: 10.5157/NEPS:SC6:11.1.0. From 2008 to 2013, NEPS data was collected as part of the Framework Program for the Promotion of Empirical Educational Research funded by the German Federal Ministry of Education and Research (BMBF). As of 2014, NEPS has been carried out by the Leibniz Institute for Educational Trajectories (LIfBi) at the University of Bamberg in cooperation with a nationwide network.

## Author Contributions

FR contributed to conceptualization, methodology, formal analysis, investigation, writing – original draft, and writing – review and editing.

## Conflict of Interest

The author declares that the research was conducted in the absence of any commercial or financial relationships that could be construed as a potential conflict of interest.

## Publisher’s Note

All claims expressed in this article are solely those of the authors and do not necessarily represent those of their affiliated organizations, or those of the publisher, the editors and the reviewers. Any product that may be evaluated in this article, or claim that may be made by its manufacturer, is not guaranteed or endorsed by the publisher.

## Footnotes

1
https://osf.io/9873s?view_only=a31efb8751274f5fa202435d055a7b04

2
https://osf.io/82m39/?view_only=cf48b92d1bec47a1b8865412ad3337fa

## References

[B1] Ajzen I. (2011). The theory of planned behaviour: reactions and reflections. *Psychol. Health* 26 1113–1127. 10.1080/08870446.2011.613995 21929476

[B2] Ajzen I. (2012). “The theory of planned behavior,” in *Handbook of Theories of Social Psychology*, Vol. 1 eds van Lange P. Kruglanski A. Higgins E. (Thousand Oaks, CA: SAGE Publications), 438–459. 10.4135/9781446249215.n22

[B3] Ajzen I. (2020). The theory of planned behavior: frequently asked questions. *Hum. Behav. Emerg. Technol.* 2 314–324. 10.1002/hbe2.195

[B4] Allison P. D. (2009). *Fixed Effects Regression Models. Quantitative Applications in the Social Sciences*, Vol. 160. Thousand Oaks, CA: SAGE.

[B5] Armitage C. J. Conner M. (2001). Efficacy of the theory of planned behaviour: a meta-analytic review. *Br. J. Soc. Psychol.* 40 471–499. 10.1348/014466601164939 11795063

[B6] Baert H. de Rick K. van Valckenborgh K. (2006). “Towards the conceptualization of ‘learning climate’,” in *Adult Education: New Routes in a New Landscape*, eds Castro R. V. D. Sancho A. V. Guimarães P. (Braga: University of Minho), 87–112.

[B7] Barrett A. O’Connell P. J. (2001). Does training generally work? The returns to in-company training. *Ind. Labor Relat. Rev.* 54 647–662. 10.1177/001979390105400307

[B8] Becker R. (2019). Economic change and continuous vocational training in the work history: a longitudinal multilevel analysis of the employees’ participation in further training and the effects on their occupational careers in Germany, 1970–2008. *Empir. Res. Vocat. Educ. Train.* 11:4. 10.1186/s40461-019-0079-x

[B9] Bellmann L. Leber U. (2019). *Bildungsökonomik.* Berlin: de Gruyter. 10.1515/9783110642315

[B10] BIBB (2020). *Datenreport zum Berufsbildungsbericht 2020: Informationen und Analysen zur Entwicklung der beruflichen Bildung [Data Report to the Vocational Training Report 2019: Information and Analysis on the Development of Vocational Education and Training].* Bonn: BIBB.

[B11] Blossfeld H.-P. Roßbach H.-G. von Maurice J. (eds) (2011). *Zeitschrift für Erziehungswissenschaft: Sonderheft 14. Education as a Lifelong Process – The German National Educational Panel Study (NEPS).* Wiesbaden: VS Verlag für Sozialwissenschaften.

[B12] BMBF (ed.) (2017). *Weiterbildungsverhalten in Deutschland 2016: Ergebnisse des Adult Education Survey (AES) [Continuing Education Behavior in Germany 2016 – Results of the Adult Education Survey (AES)].* Bielefeld: wbv Media. 10.3278/85/0016w

[B13] BMBF (ed.) (2019). *Weiterbildungsverhalten in Deutschland 2018: Ergebnisse des Adult Education Survey – AES-Trendbericht [Continuing Education Behavior in Germany 2018 – Results of the Adult Education Survey (AES)].* Bielefeld: wbv Media.

[B14] Boeren E. (2011). Participation in adult education: attitudes and barriers. *U.S. China Educ. Rev. A* 1 369–382.

[B15] Boeren E. (2017). Understanding adult lifelong learning participation as a layered problem. *Stud. Contin. Educ.* 39 161–175. 10.1080/0158037X.2017.1310096

[B16] Boeren E. Nicaise I. Baert H. (2010). Theoretical models of participation in adult education: the need for an integrated model. *Int. J. Lifelong Educ.* 29 45–61. 10.1080/02601370903471270

[B17] Bosnjak M. Ajzen I. Schmidt P. (2020). The theory of planned behavior: selected recent advances and applications. *Eur. J. Psychol.* 16 352–356. 10.5964/ejop.v16i3.3107 33680187 PMC7909498

[B18] CEDEFOP (2014). *Policy Handbook: Access to and Participation in Continuous Vocational Education and Training (CVET) in Europe (CEDEFOP Working Paper No. 25).* Luxembourg: Publications Office of the European Union.

[B19] CEDEFOP (2015). *Job-Related Adult Learning and Continuing Vocational Training in Europe: A Statistical Picture. CEDEFOP Research Paper: Vol 48.* Luxembourg: Publications Office of the European Union. 10.2801/392276

[B20] Cross K. P. (1981). *Adults as Learners.* Hoboken, NJ: Jossey-Bass.

[B21] de Grip A. Sauermann J. (2013). The effect of training on productivity: the transfer of on-the-job training from the perspective of economics. *Educ. Res. Rev.* 8 28–36. 10.1016/j.edurev.2012.05.005

[B22] Denninger A. Kahl R. Präßler S. (2020). *Individuumsbezogene Zeitbudgetstudie [Study on Individual Time Budgets].* Wiesbaden: Springer Fachmedien Wiesbaden. 10.1007/978-3-658-27501-3

[B23] Ebner C. Ehlert M. (2018). Weiterbilden und Weiterkommen? Non-formale berufliche Weiterbildung und Arbeitsmarktmobilität in Deutschland [does further education lead to career advancement? Non-formal further training and labour market mobility in Germany]. *Köln. Z. Soziol. Sozialpsychol.* 70 213–235. 10.1007/s11577-018-0518-x

[B24] Fishbein M. Ajzen I. (2010). *Predicting and Changing Behavior: The Reasoned Action Approach.* Hove: Psychology Press.

[B25] Fleuren B. P. de Grip A. Kant I. Zijlstra F. R. (2020). Time equals money? A randomized controlled field experiment on the effects of four types of training vouchers on training participation. *J. Vocat. Behav.* 118:103403. 10.1016/j.jvb.2020.103403

[B26] Gorges J. Kandler C. (2012). Adults’ learning motivation: expectancy of success, value, and the role of affective memories. *Learn. Individ. Differ.* 22 610–617. 10.1016/j.lindif.2011.09.016

[B27] Grund C. Martin J. (2012). Determinants of further training – evidence for Germany. *Int. J. Hum. Resour. Manage.* 23 3536–3558. 10.1080/09585192.2011.654347

[B28] Hurtz G. M. Williams K. J. (2009). Attitudinal and motivational antecedents of participation in voluntary employee development activities. *J. Appl. Psychol.* 94 635–653. 10.1037/a0014580 19450004

[B29] Kaufmann K. Widany S. (2013). Berufliche Weiterbildung – Gelegenheits- und Teilnahmestrukturen [Continuing vocational education and training – opportunity structures and participation]. *Z. Erziehungswiss.* 16 29–54. 10.1007/s11618-013-0338-8

[B30] Klein C. C. (2007). *The Economics of Time As A Resource. Working Paper Series.* Murfreesboro, TN: Middle Tennessee State University, Department of Economics and Finance.

[B31] Kyndt E. Baert H. (2013). Antecedents of employees’ involvement in work-related learning. *Rev. Educ. Res.* 83 273–313. 10.3102/0034654313478021

[B32] Kyndt E. Govaerts N. Dochy F. Baert H. (2011). The learning intention of low-qualified employees: a key for participation in lifelong learning and continuous training. *Vocat. Learn.* 4 211–229. 10.1007/s12186-011-9058-5

[B33] La Barbera F. Ajzen I. (2020). Control interactions in the theory of planned behavior: rethinking the role of subjective norm. *Eur. J. Psychol.* 16 401–417. 10.5964/ejop.v16i3.2056 33680190 PMC7909507

[B34] La Barbera F. Ajzen I. (2021). Moderating role of perceived behavioral control in the theory of planned behavior: a preregistered study. *J. Theor. Soc. Psychol.* 5 35–45. 10.1002/jts5.83

[B35] Leyretana K. Trinidad J. E. (2021). Predicting or preventing lifelong learning? The role of employment, time, cost, and prior achievement. *J. Adult Contin. Educ.* 1–16. 10.1177/14779714211054555

[B36] Lischewski J. Seeber S. Wuttke E. Rosemann T. (2020). What influences participation in non-formal and informal modes of continuous vocational education and training? An analysis of individual and institutional influencing factors. *Front. Psychol.* 11:534485. 10.3389/fpsyg.2020.534485 33343436 PMC7744883

[B37] Maurer T. J. Weiss E. M. Barbeite F. G. (2003). A model of involvement in work-related learning and development activity: the effects of individual, situational, motivational, and age variables. *J. Appl. Psychol.* 88 707–724. 10.1037/0021-9010.88.4.707 12940410

[B38] McDermott M. S. Oliver M. Svenson A. Simnadis T. Beck E. J. Coltman T. (2015). The theory of planned behaviour and discrete food choices: a systematic review and meta-analysis. *Int. J. Behav. Nutr. Phys. Act.* 12 1–11. 26715190 10.1186/s12966-015-0324-z PMC4696173

[B39] Nguyen T.-M. Nham P. T. Hoang V.-N. (2019). The theory of planned behavior and knowledge sharing. *VINE J. Inf. Knowl. Manage. Syst.* 49 76–94. 10.1108/VJIKMS-10-2018-0086

[B40] OECD (2013). *OECD Skills Outlook 2013: First Results from the Survey of Adult Skills.* Paris: OECD Publishing. 10.1787/9789264204256-en

[B41] Opp K.-D. (2019). “Can attitude theory improve rational choice theory or vice versa?” in *Einstellungen und Verhalten in der Empirischen Sozialforschung [Attitudes and Behavior in Empirical Social Research]*, eds Mayerl J. Krause T. Wahl A. Wuketich M. (Wiesbaden: Springer Fachmedien Wiesbaden), 65–95. 10.1007/978-3-658-16348-8_4

[B42] Parkinson J. David P. Rundle-Thiele S. (2017). Self-efficacy or perceived behavioural control: which influences consumers’ physical activity and healthful eating behaviour maintenance? *J. Consum. Behav.* 16 413–423. 10.1002/cb.1641

[B43] Rabe-Hesketh S. Skrondal A. (2012). *Multilevel and Longitudinal Modeling Using Stata: Categorical Responses, Counts, and Survival*. Vol. 2. College Station, TX: Stata Press.

[B44] Rubenson K. Desjardins R. (2009). The impact of welfare state regimes on barriers to participation in adult education. *Adult Educ. Q.* 59 187–207. 10.1177/0741713609331548

[B45] Rubin D. B. (1987). *Multiple Imputation for Nonresponse in Surveys.* Hoboken, NJ: John Wiley & Sons, Inc. 10.1002/9780470316696

[B46] Ruhose J. Thomsen S. L. Weilage I. (2020). “Work-related training and subjective well-being. Estimating the effect of training participation on satisfaction, worries, and health in Germany,” in *Monetäre und nicht Monetäre Erträge von Weiterbildung [Monetary and Non-Monetary Returns from Continuing Education]*, Vol. 7 eds Schrader J. Ioannidou A. Blossfeld H.-P. (Wiesbaden: Springer Fachmedien Wiesbaden), 107–144. 10.1007/978-3-658-25513-8_5

[B47] Rüter F. Martin A. (2021). How do the timing and duration of courses affect participation in adult learning and education? A panel analysis. *Adult Educ. Q.* 1–23. 10.1177/07417136211019032

[B48] Rüter F. Martin A. Schrader J. (2020). Educational leave as a time resource for participation in adult learning and education (ALE). *Front. Psychol.* 10:2977. 10.3389/fpsyg.2019.02977 32038373 PMC6987466

[B49] Saar E. Räis M. L. (2017). Participation in job-related training in European countries: the impact of skill supply and demand characteristics. *J. Educ. Work* 30 531–551. 10.1080/13639080.2016.1243229

[B50] Schmidt-Lauff S. (2008). *Zeit für Bildung im Erwachsenenalter: Interdisziplinäre und empirische Zugänge [Time for Education in Adulthood: Interdisciplinary and Empirical Approaches] (1. Auflage). Internationale Hochschulschriften: Band 509.* Münster: Waxmann.

[B51] Schmidt-Lauff S. Bergamini R. (2017). “The modern phenomenon of adult learning and professional time-sensitivity – a temporal, comparative approach contrasting Italy and Germany,” in *Adult Learning and Education in International Contexts: Future Challenges for its Professionalization*, eds Egetenmeyer R. Schmidt-Lauff S. Boffo V. (Bern: Peter Lang Edition), 147–159.

[B52] Schunck R. (2013). Within and between estimates in random-effects models: advantages and drawbacks of correlated random effects and hybrid models. *Stata J.* 13 65–76. 10.1177/1536867X1301300105

[B53] Schunck R. Perales F. (2017). Within- and between-cluster effects in generalized linear mixed models: a discussion of approaches and the xthybrid command. *Stata J.* 17 89–115. 10.1177/1536867X1701700106

[B54] Sellin B. Elson-Rogers S. (2003). *Engaging Individuals in Lifelong Learning: Mobilising Resources, Time and Money: Lifelong Learning: Thematic Workshop Report (CEDEFOP Working Paper).* Thessaloniki: CEDEFOP.

[B55] Sheeran P. (2002). Intention-behavior relations: a conceptual and empirical review. *Eur. Rev. Soc. Psychol.* 12 1–36. 10.1080/14792772143000003

[B56] Sheeran P. Webb T. L. (2016). The Intention-behavior gap. *Soc. Pers. Psychol. Compass* 10 503–518. 10.1111/spc3.12265

[B57] Siegfried C. Berger J. (2020). Perspectives on participation in continuous vocational education training – an interview study. *Front. Psychol.* 11:1096. 10.3389/fpsyg.2020.01096 32695037 PMC7338917

[B58] Sussman R. Gifford R. (2019). Causality in the theory of planned behavior. *Pers. Soc. Psychol. Bull.* 45 920–933. 10.1177/0146167218801363 30264655

[B59] Valois P. Talbot D. Bouchard D. Renaud J.-S. Caron M. Canuel M. (2020). Using the theory of planned behavior to identify key beliefs underlying heat adaptation behaviors in elderly populations. *Popul. Environ.* 41 480–506. 10.1007/s11111-020-00347-5

[B60] Walter M. Müller N. (2014). Weiterbildungsbeteiligung und individuelle nutzenerwartungen [Participation in continuing education and expected individual benefits]. *Berufs and Wirtschaftspädagogik Online* 26, 1–19.

[B61] Yang-Wallentin F. Schmidt P. Davidov E. Bamberg S. (2004). Is there any interaction effect between intention and perceived behavioral control? *Methods Psychol. Res. Online* 8 127–157.

